# Supplementary material for: Ume6-dependent pathways of morphogenesis and biofilm formation in Candida auris
Source: Microbiol Spectr. 2024 Sep 19;12(11):e01531-24. doi: 10.1128/spectrum.01531-24 (PMC11537075; doi:10.1128/spectrum.01531-24)
Supplement: Supplemental figures — Fig. S1 to S27. [file spectrum.01531-24-s0001.pdf]

Figure S1

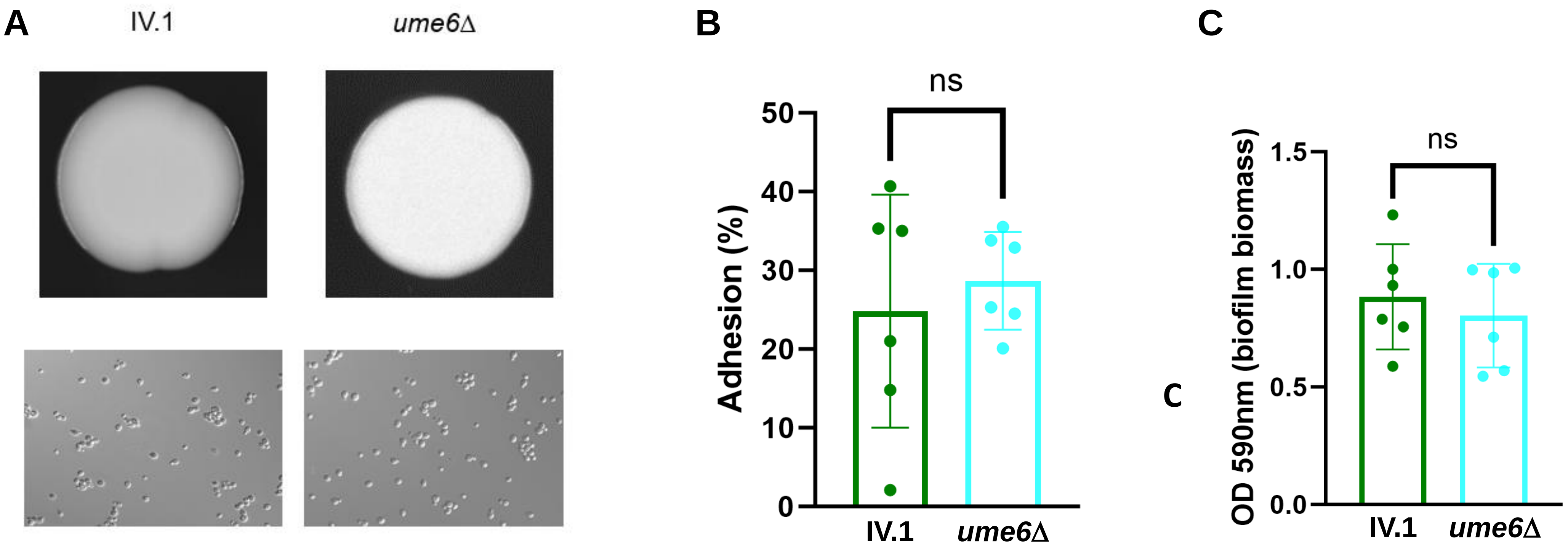

Fig S_ Deletion of *UME6* has no impact on morphology, biofilm formation and adhesion
Comparison of the *UME6* deletion strain (*ume6*Δ) and its background wild-type strain IV.1
A Top panel : macroscopic pictures of the colonies after overnight culture on solid YEPD at 37°C
Bottom panel : Differential interference contrast (DIC) microscopy after overnight growth in liquid YEPD at 37°C. The images were captured at magnification 630x with a ZEISS Axiocam 305 (Carl Zeiss AG, Oberkochen, Germany) using Zen software.
B Percentage of adhesion of yeast cells to fluorescent polystyrene microspheres (Y axis). Data represents mean + standard deviation of technical triplicates and biological duplicates. Statistical analysis was performed using unpaired T-test with significant p-value defined as ≤ 0.05 (* ≤ 0.05, ** ≤ 0.01, *** ≤ 0.001, **** ≤ 0.0001).
C Absorbance, which is representative of the biomass of adherent cells to polystyrene surface, measured at 590 nm after 24 hours incubation (Y axis). Data represents mean + standard deviation of technical triplicates and biological duplicates. Statistical analysis was performed using unpaired T-test with significant p-value defined as ≤ 0.05 (* ≤ 0.05, ** ≤ 0.01, *** ≤ 0.001, **** ≤ 0.0001).

Figure S2

IV.1 | *UME6^HA* | *UME6^HA/als4498Δ* | *UME6^HA/scf1Δ* | *UME6^HA/hgc1Δ*

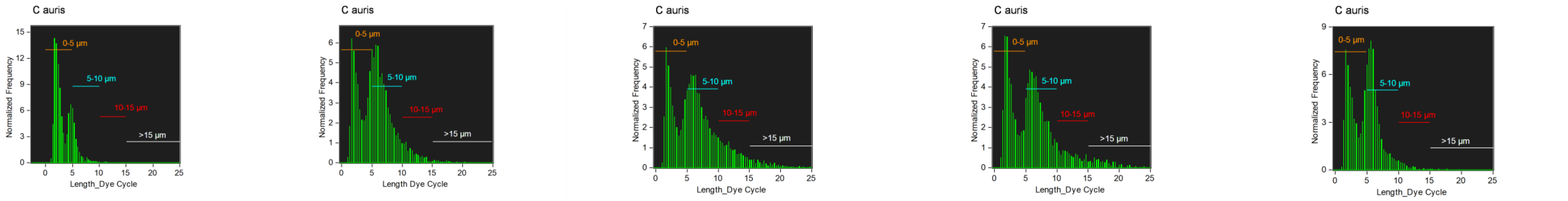

IV.1 — Length_Dye Cycle

Population	Count	%Gated	Mean
C auris & Focus	6930	100	3.329
0-5 µm & C auris & Focus	5939	85.7	2.832
5-10 µm & C auris & Focus	965	13.9	6.173
10-15 µm & C auris & Focus	26	0.38	11.26
>15 µm & C auris & Focus	0	0	NaN

UME6^HA — Length Dye Cycle

Population	Count	%Gated	Mean
C auris & Focus	4390	100	5.627
0-5 µm & C auris & Focus	1985	45.2	3.134
5-10 µm & C auris & Focus	2076	47.3	6.925
10-15 µm & C auris & Focus	287	6.54	11.75
>15 µm & C auris & Focus	41	0.93	17.13

UME6^HA/als4498Δ — Length_Dye Cycle

Population	Count	%Gated	Mean
C auris & Focus	7586	100	6.668
0-5 µm & C auris & Focus	2930	38.6	2.966
5-10 µm & C auris & Focus	3344	44.1	7.129
10-15 µm & C auris & Focus	979	12.9	12.2
>15 µm & C auris & Focus	326	4.3	18.13

UME6^HA/scf1Δ — Length_Dye Cycle

Population	Count	%Gated	Mean
C auris & Focus	2401	100	6.404
0-5 µm & C auris & Focus	972	40.5	2.765
5-10 µm & C auris & Focus	1082	45.1	7.07
10-15 µm & C auris & Focus	222	9.25	12.35
>15 µm & C auris & Focus	123	5.12	18.14

UME6^HA/hgc1Δ — Length_Dye Cycle

Population	Count	%Gated	Mean
C auris & Focus	6028	100	4.858
0-5 µm & C auris & Focus	3165	52.5	3.057
5-10 µm & C auris & Focus	2677	44.4	6.463
10-15 µm & C auris & Focus	162	2.69	11.62
>15 µm & C auris & Focus	24	0.4	17.81

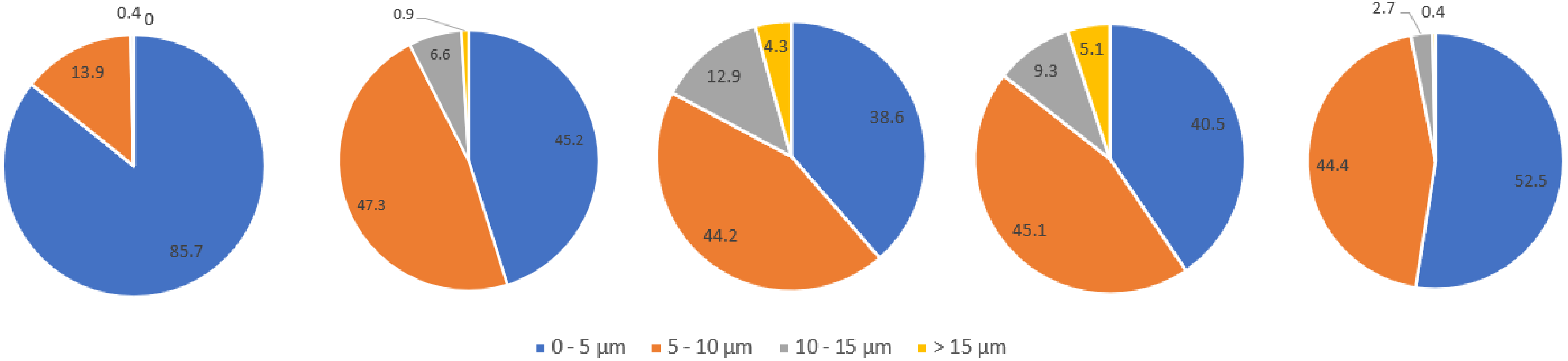

Cell length analysis by imaging flow cytometry for the strains IV.1, *UME6^HA*, *UME6^HA/als4498Δ*, *UME6^HA/scf1Δ* and *UME6^HA/hgc1Δ*

Top: quantification of length of the yeast cells subdivided in 4 subpopulations (0-5 µm, 5-10 µm, 10-15 µm and > 15 µm) on the X axis and frequency of each event on the Y axis.

Bottom: graphical representation with sector diagrams of the repartition of each length's subpopulation

Figure S3

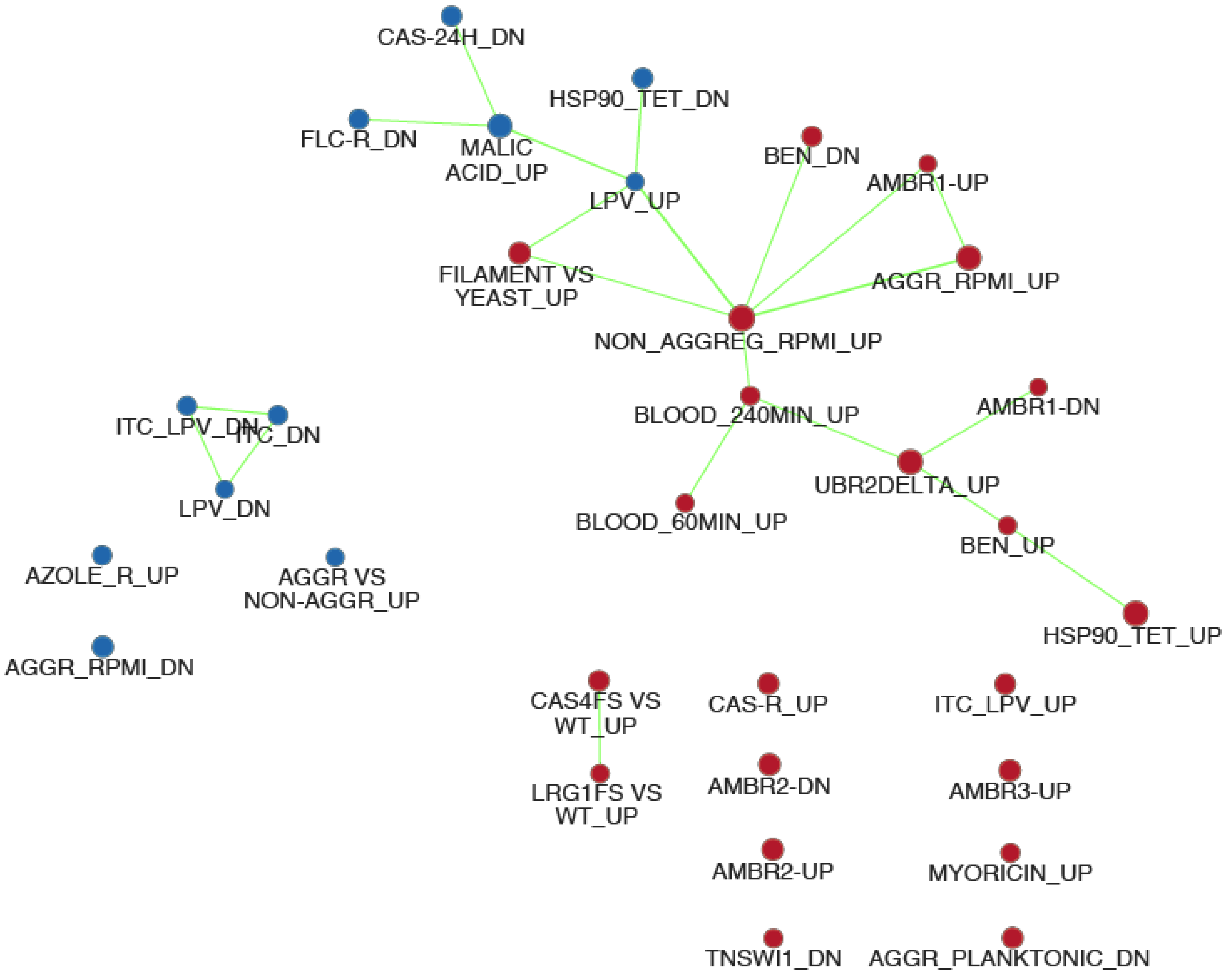

GSEA analysis of Ume6-regulated genes in *C. auris*. Red nodes represent enriched gene lists in upregulated genes from the GSEA. Blue nodes represent enriched gene lists in downregulated genes from the GSEA. Nodes are connected by edges (green lines) when overlaps exist between nodes. The size of nodes reflects the total number of genes that are connected by edges to neighboring nodes. Edge thickness reflects the level of confidence between nodes. Labels of nodes indicate specific classes of up- and downregulated genes listed in the Cauris.gmt file (in File S1).

Figure S4

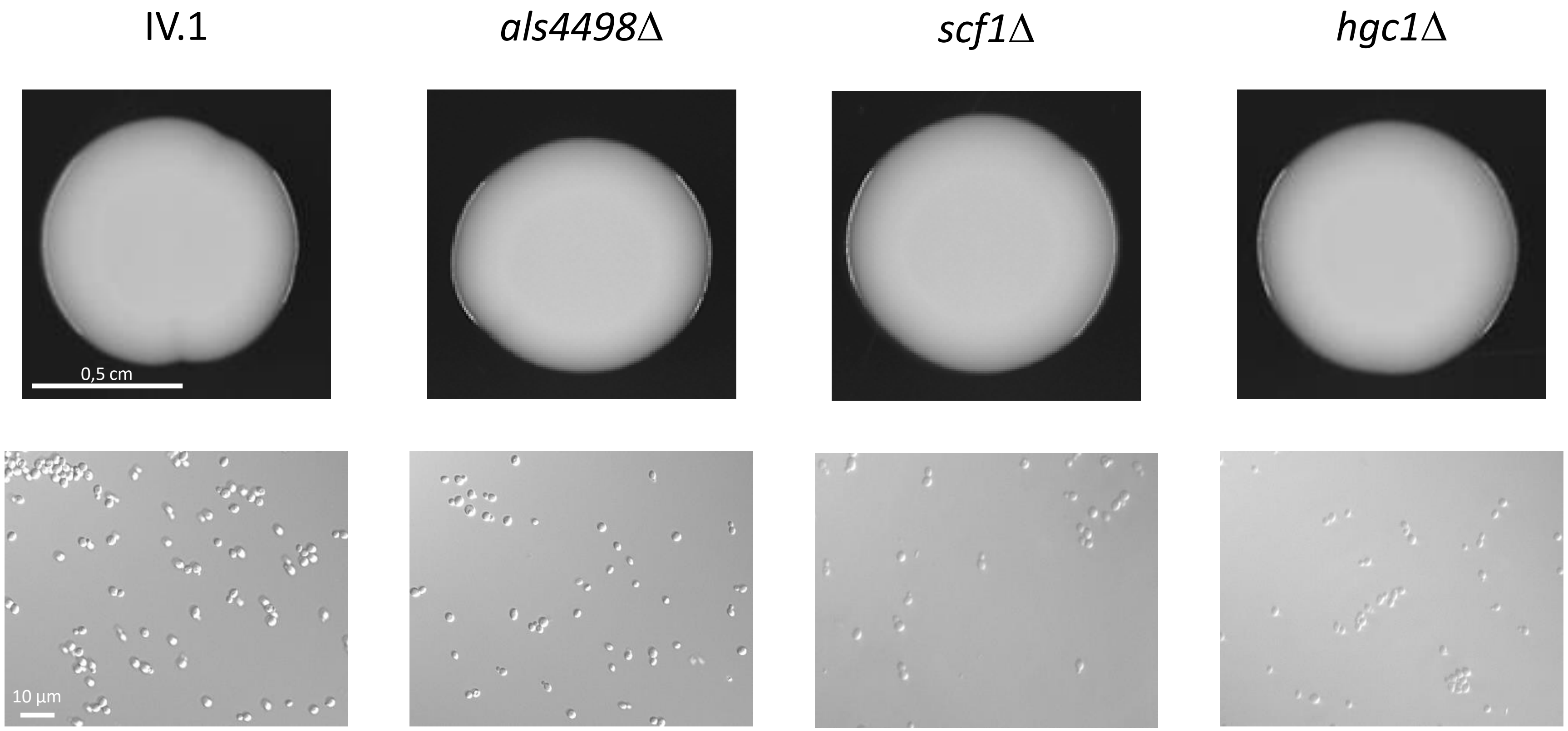

Phenotypic aspects of the IV.1, *als4498*Δ, *scf1*Δ and *hgc1*Δ strains
Top row: colonies on yeast peptone dextrose (YEPD) agar plates, after 4 days of growth at 37°C
Bottom row: differential interference contrast (DIC) microscopy at 630x magnification, after overnight culture at 37°C in liquid YEPD

Figure S5

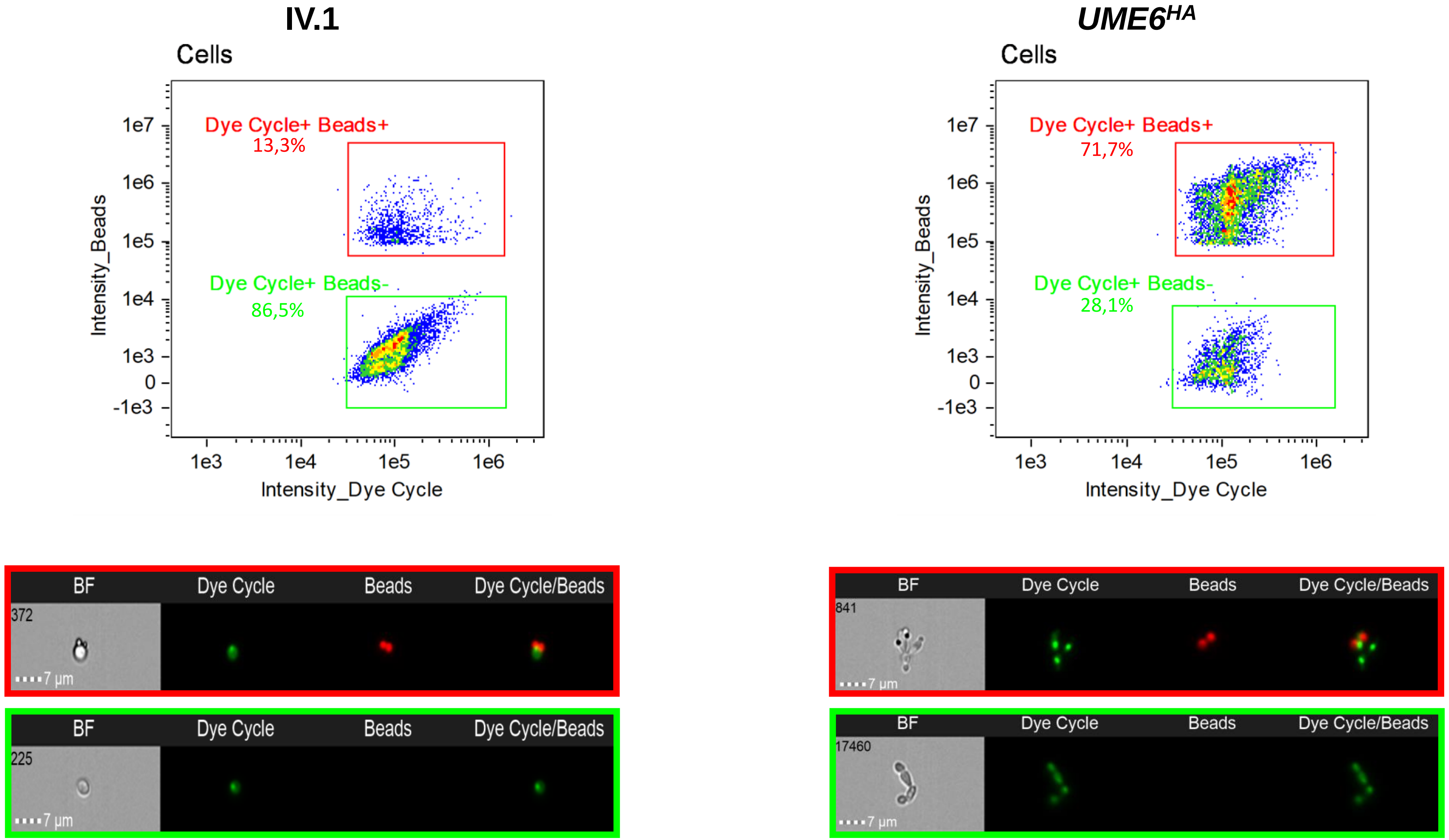

Adhesion assay by imaging flow cytometry for the strains IV.1 and *UME6^{HA}*.

Top: graphical representation of the intensity of the red fluorescent signal of the beads (microspheres, y-axis) and the intensity of the fluorescent DyeCycle™ Green stain of the yeast cells (x-axis). The subpopulation of yeasts that have attached the beads is delimited by the open red square (Dye cycle + Beads+). The subpopulation of yeasts that have not attached the beads is delimited by the open green square (Dye cycle + Beads-).

Bottom: representative pictures of each population. Representative images are shown in the bottom rows including bright-field (BF, grey), yeast cells (DyeCycle™, green), beads (FluoSpheres™, red) and a composite overlay of yeast cells and beads. Scale bars represent 7 µm.

Figure S6

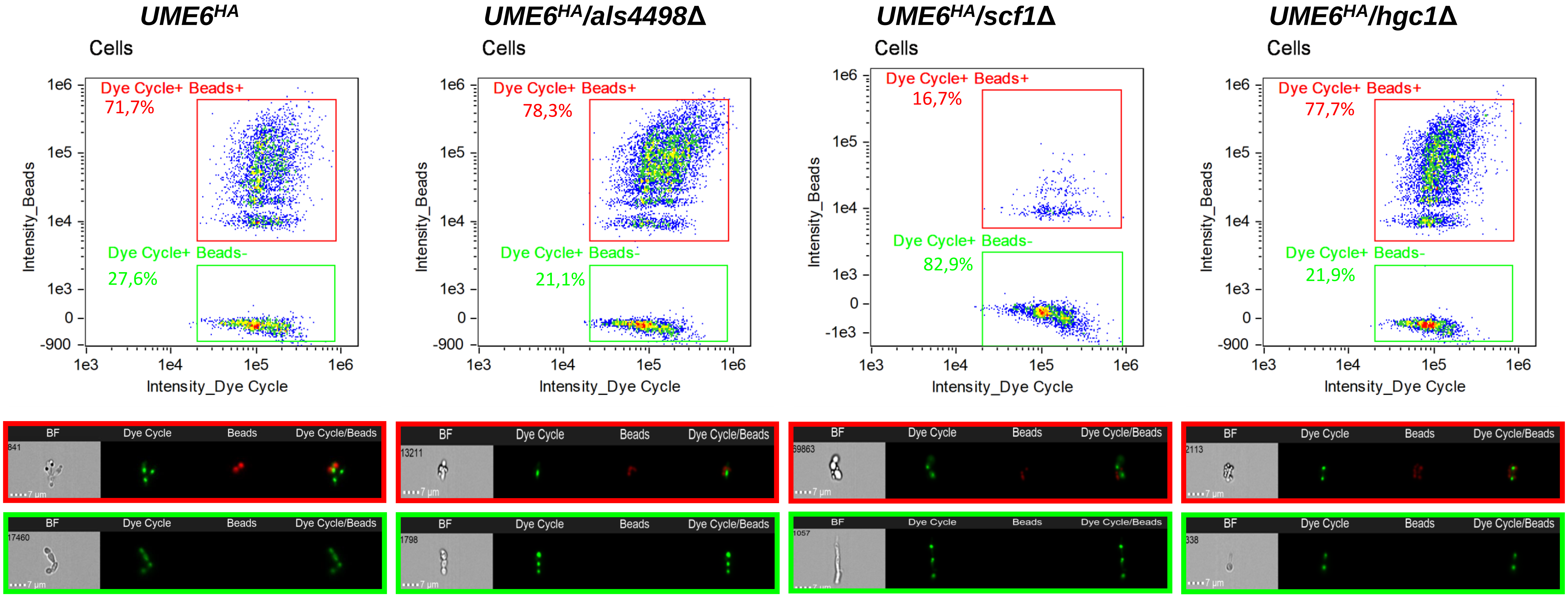

Adhesion assay by imaging flow cytometry for the strains *$UME6^{HA}$*, *$UME6^{HA}$/als4498Δ*, *$UME6^{HA}$/scf1Δ* and *$UME6^{HA}$/hgc1Δ*.

Top: graphical representation of the intensity of the red fluorescent signal of the beads (microspheres, y-axis) and the intensity of the fluorescent DyeCycle™ Green stain of the yeast cells (x-axis). The subpopulation of yeasts that have attached the beads is delimited by the open red square (Dye cycle + Beads+). The subpopulation of yeasts that have not attached the beads is delimited by the open green square (Dye cycle + Beads-).

Bottom: representative pictures of each population. Representative images are shown in the bottom rows including bright-field (BF, grey), yeast cells (DyeCycle™, green), beads (FluoSpheres™, red) and a composite overlay of yeast cells and beads. Scale bars represent 7 µm.

Figure S7

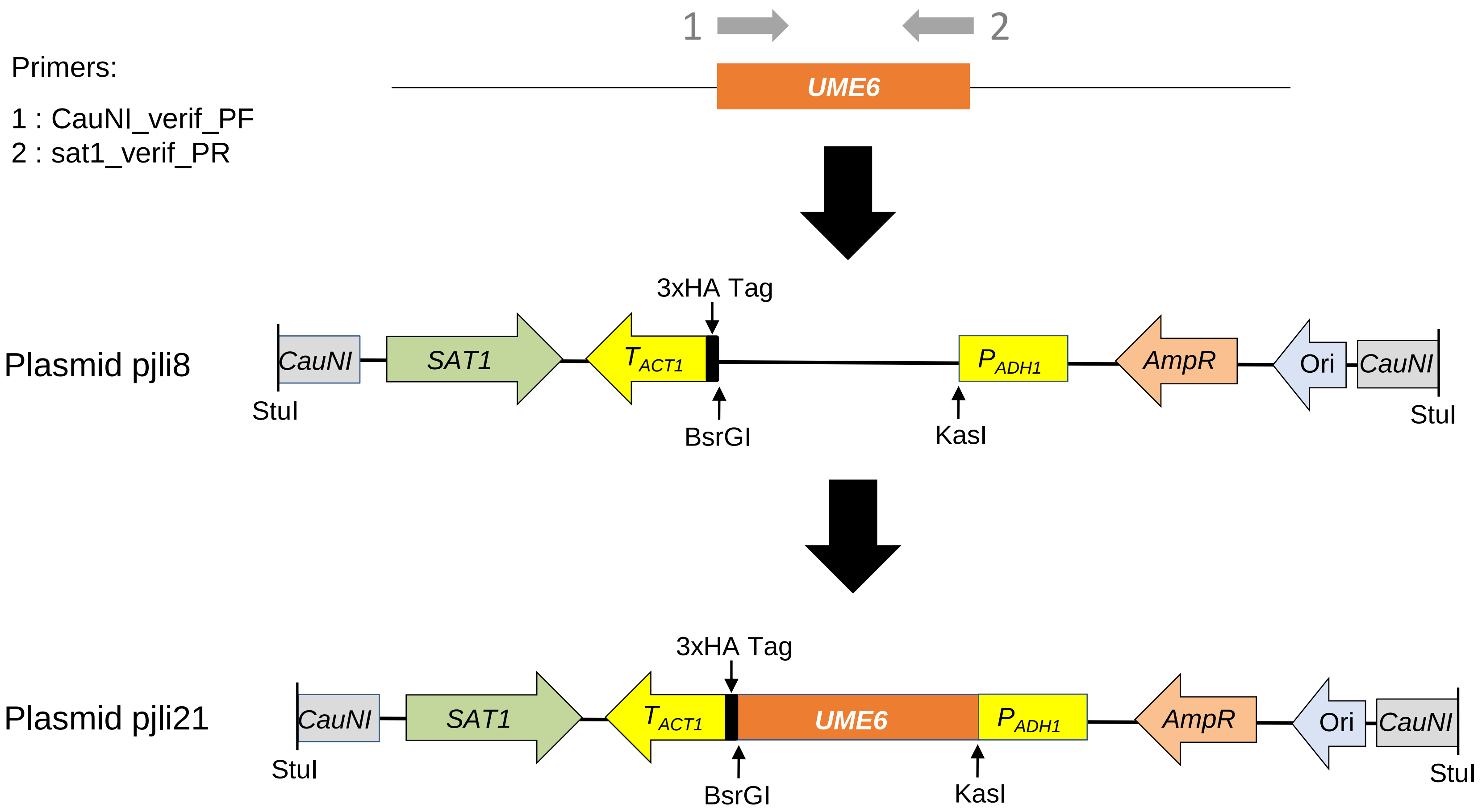

Construct for Ume6 hyperactivation (*UME6*^{HA} strain). The *UME6* PCR product was cloned at KasI and BsrGI sites in plasmid pjli8 containing the promotor P_{ADH1}, the 3xHa Tag, the terminator of T_{ACT1}, the SAT1 cassette (nourseothricin resistance) and the *C. auris* neutral site *CauNI*. The resulting plasmid was linearized by Stu1.

Figure S8

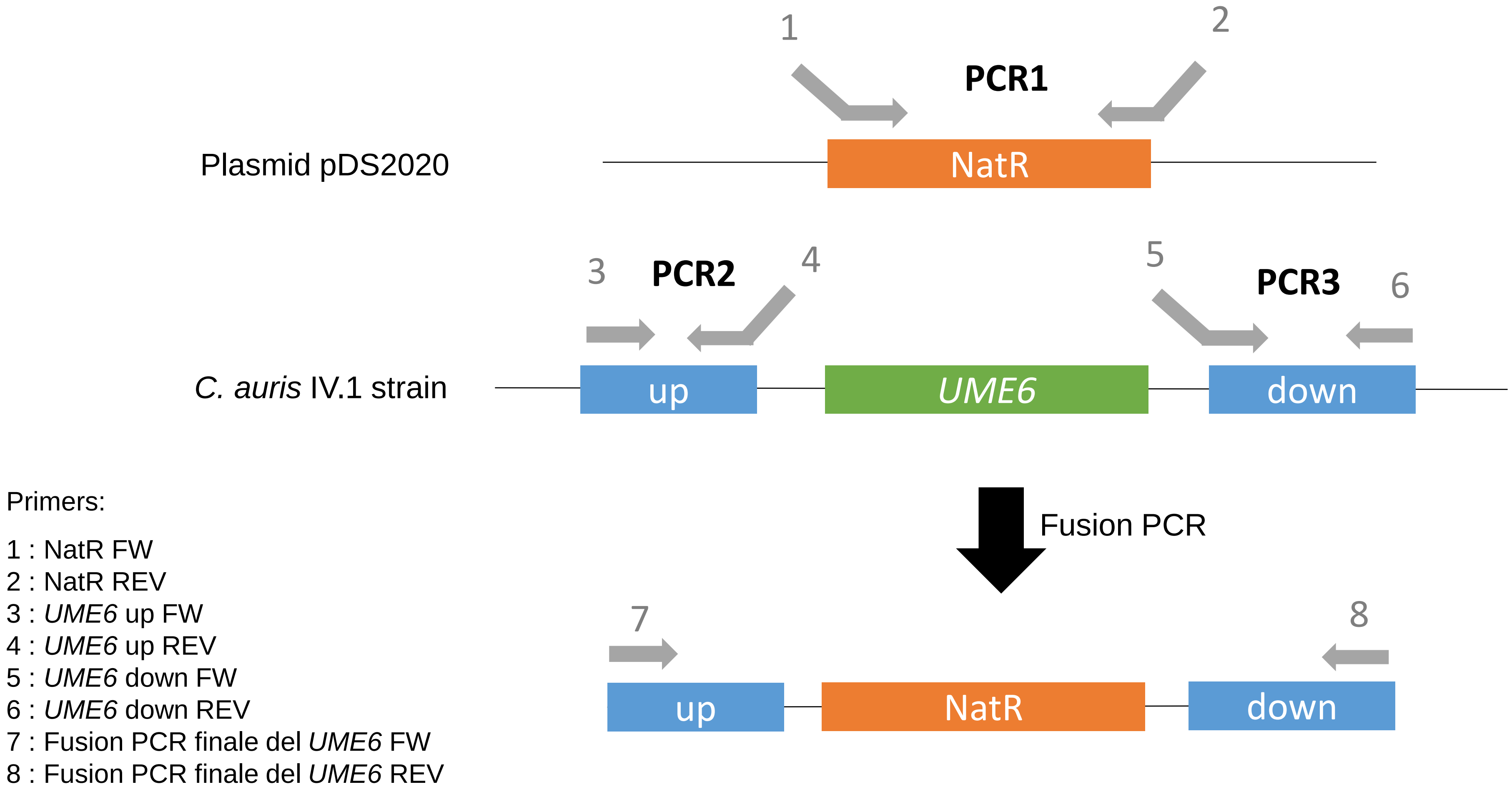

Construct for *UME6* deletion (*ume6*Δ strain). The construct was obtained by fusion PCR of the 500 bp upstream region (up) of *UME6*, the NatR cassette and the 500 bp downstream region (down) of *UME6*.

Figure S9

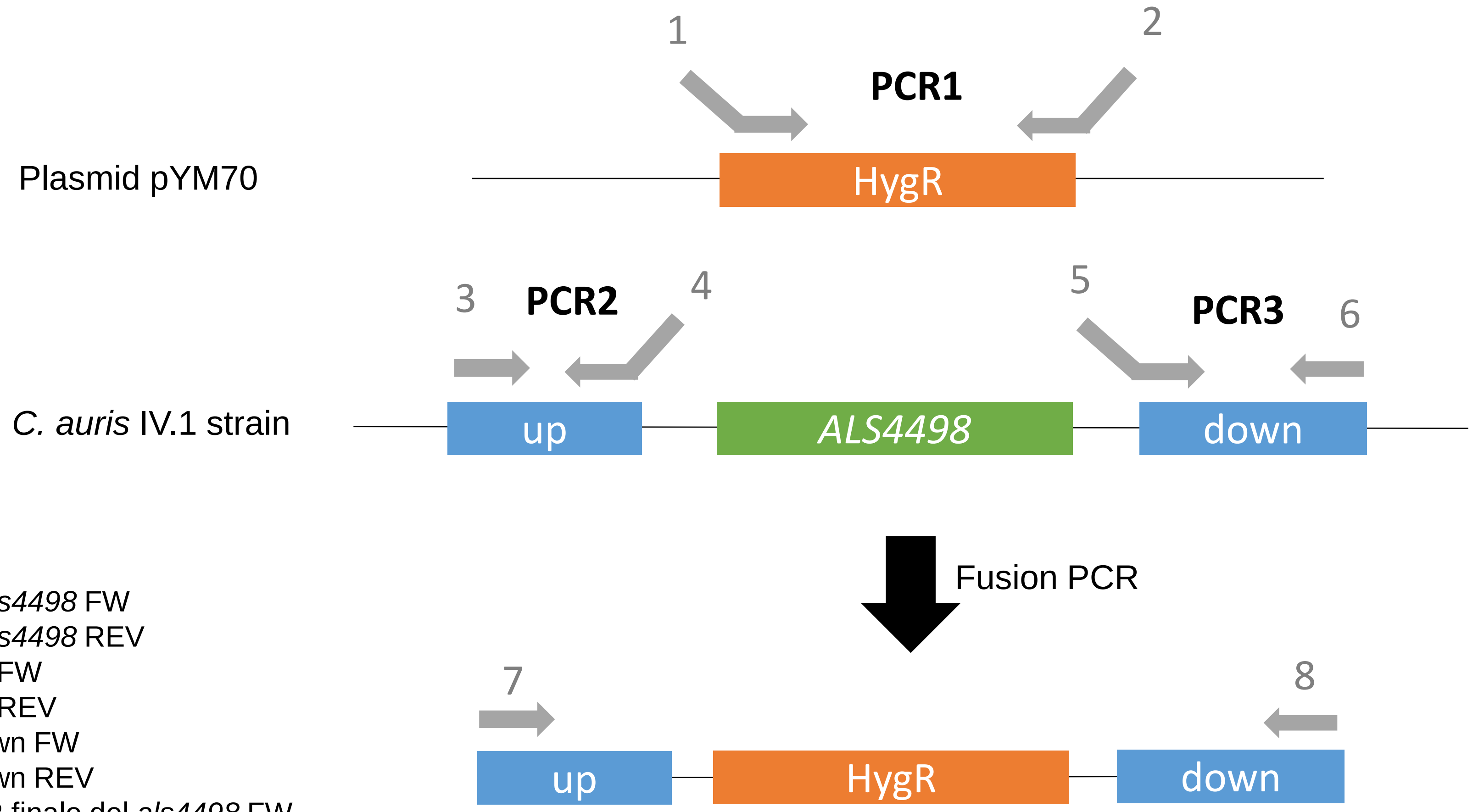

Primers:

1 : HygR del *als4498* FW
2 : HygR del *als4498* REV
3 : *als4498* up FW
4 : *als4498* up REV
5 : *als4498* down FW
6 : *als4498* down REV
7 : Fusion PCR finale del *als4498* FW
8 : Fusion PCR finale del *als4498* REV

Construct for *ALS4498* deletion (*als4498*Δ and *UME6*^{HA}/*als4498*Δ strains). The construct was obtained by fusion PCR of the 500 bp upstream region (up) of *als4498*, the NatR cassette and the 500 bp downstream region (down) of *ALS4498*.

Figure S10

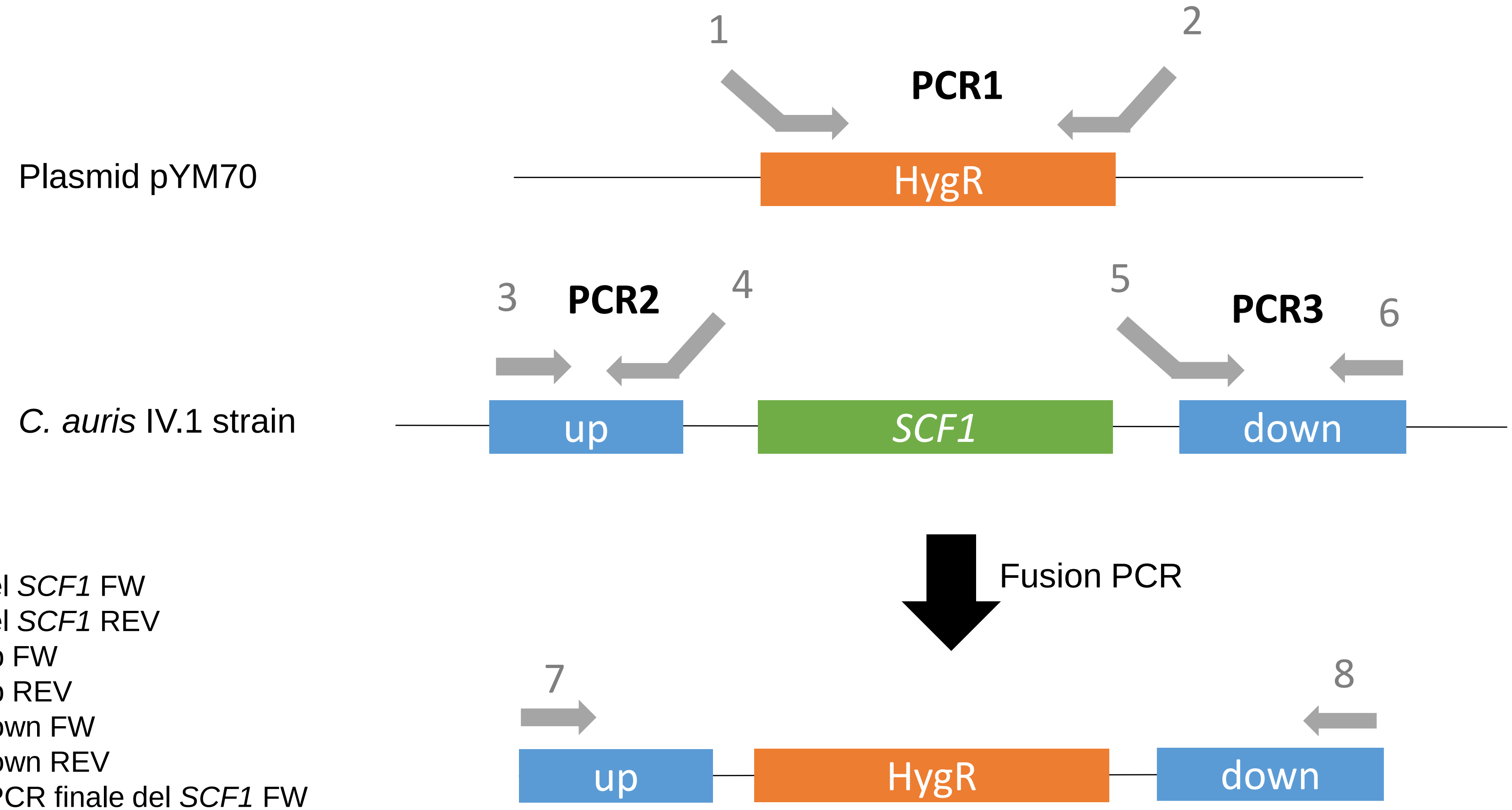

Primers:

1 : HygR del *SCF1* FW
2 : HygR del *SCF1* REV
3 : *SCF1* up FW
4 : *SCF1* up REV
5 : *SCF1* down FW
6 : *SCF1* down REV
7 : Fusion PCR finale del *SCF1* FW
8 : Fusion PCR finale del *SCF1* REV

Construct for *SCF1* deletion (*scf1*Δ and *UME6*^{HA}*/scf1*Δ strains). The construct was obtained by fusion PCR of the 500 bp upstream region (up) of *SCF1*, the NatR cassette and the 500 bp downstream region (down) of *SCF1*.

Figure S11

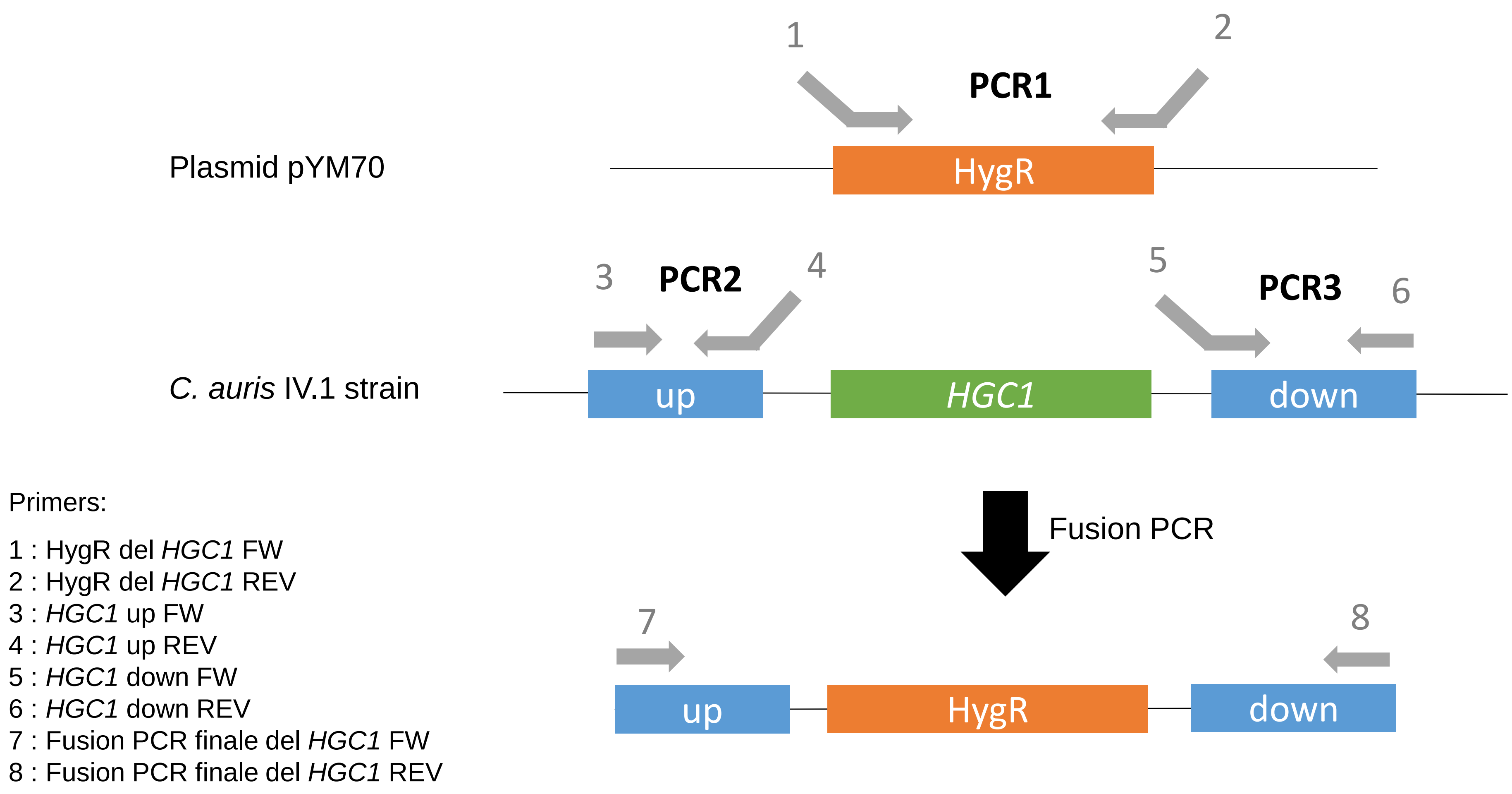

Construct for *HGC1* deletion (*hgc1*Δ and *UME6*^{HA}/*hgc1*Δ strains). The construct was obtained by fusion PCR of the 500 bp upstream region (up) of *HGC1*, the NatR cassette and the 500 bp downstream region (down) of *HGC1*.

Figure S12

Primers:

1 : *UME6* 5’ FW
2 : *UME6* 5’ REV

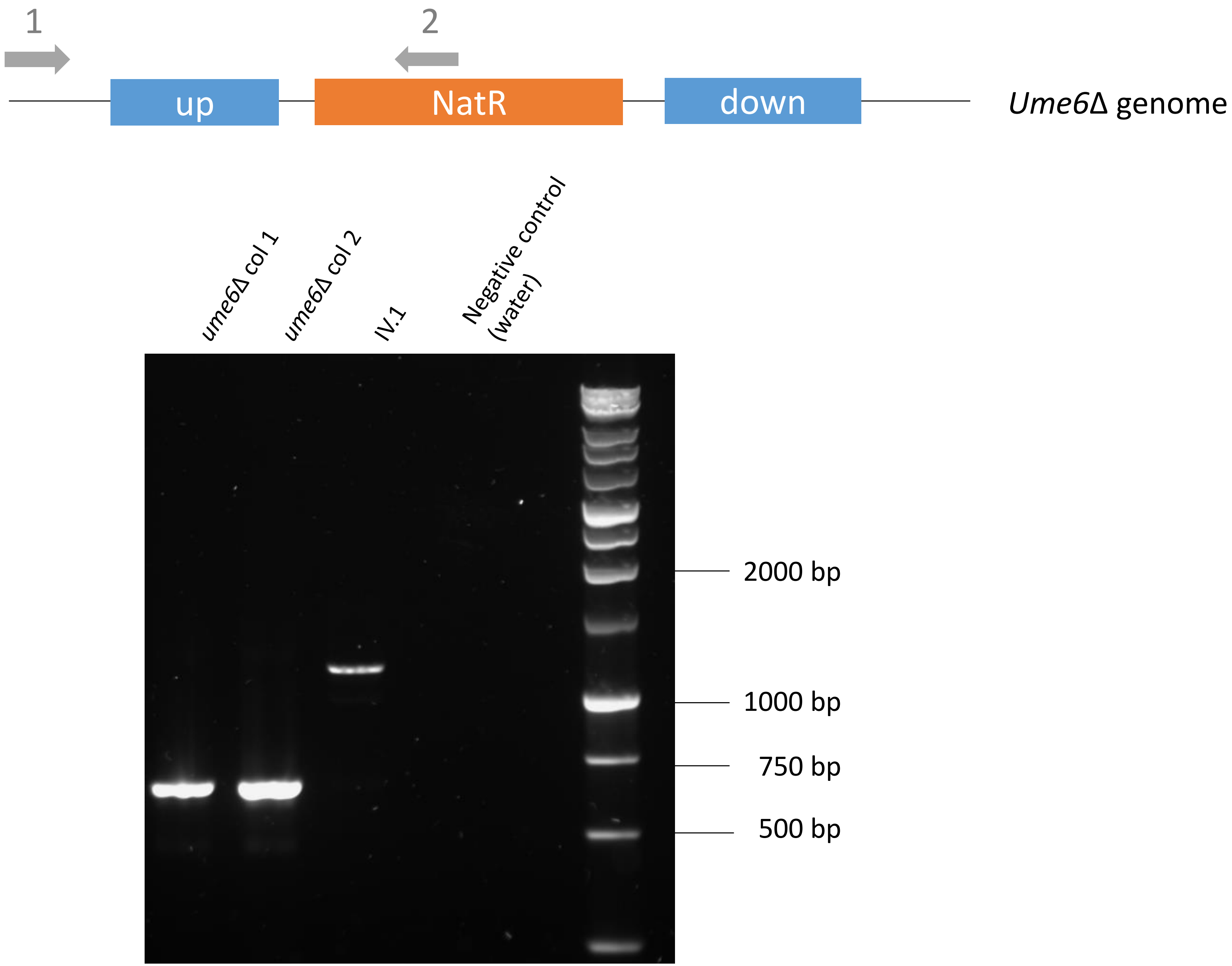

Verification of the deletion of *UME6* in the *ume6*Δ strain. An approximately 700 bp fragment was amplified with a forward primer located upstream of the “up” region and a reverse primer in *NatR*. As shown on the electrophoresis gel, the PCR product was present in the mutant strain and absent in the IV.1 strain.

Figure S13

Primers:

1 : *UME6* 3’ FW
2 : *UME6* 3’ REV

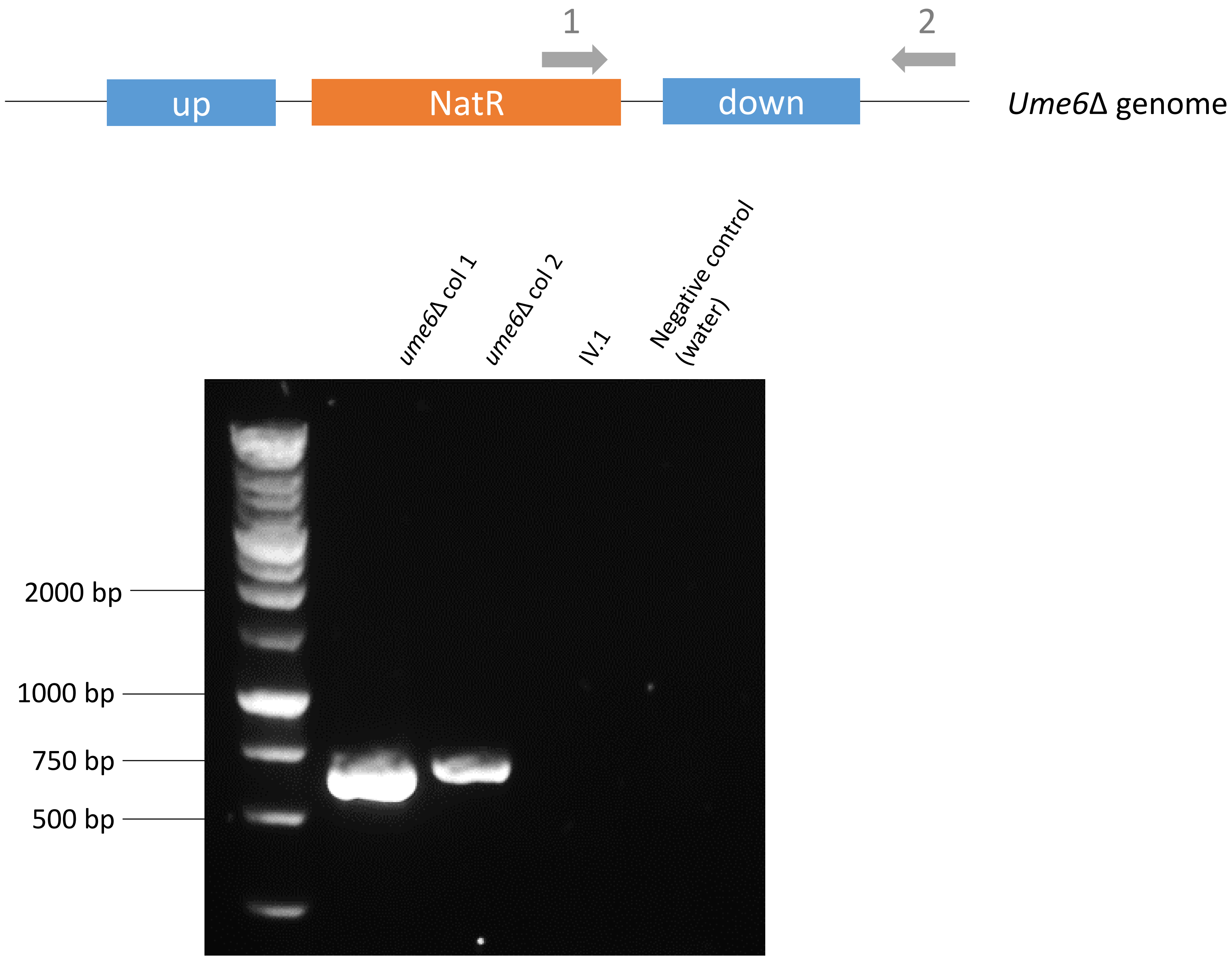

Verification of the deletion of *UME6* in the *ume6*Δ strain. An approximately 700 bp fragment was amplified with a forward primer in *NatR* and a reverse primer located downstream of the “down” region. As shown on the electrophoresis gel, the PCR product was present in the mutant strain and absent in the IV.1 strain.

Figure S14

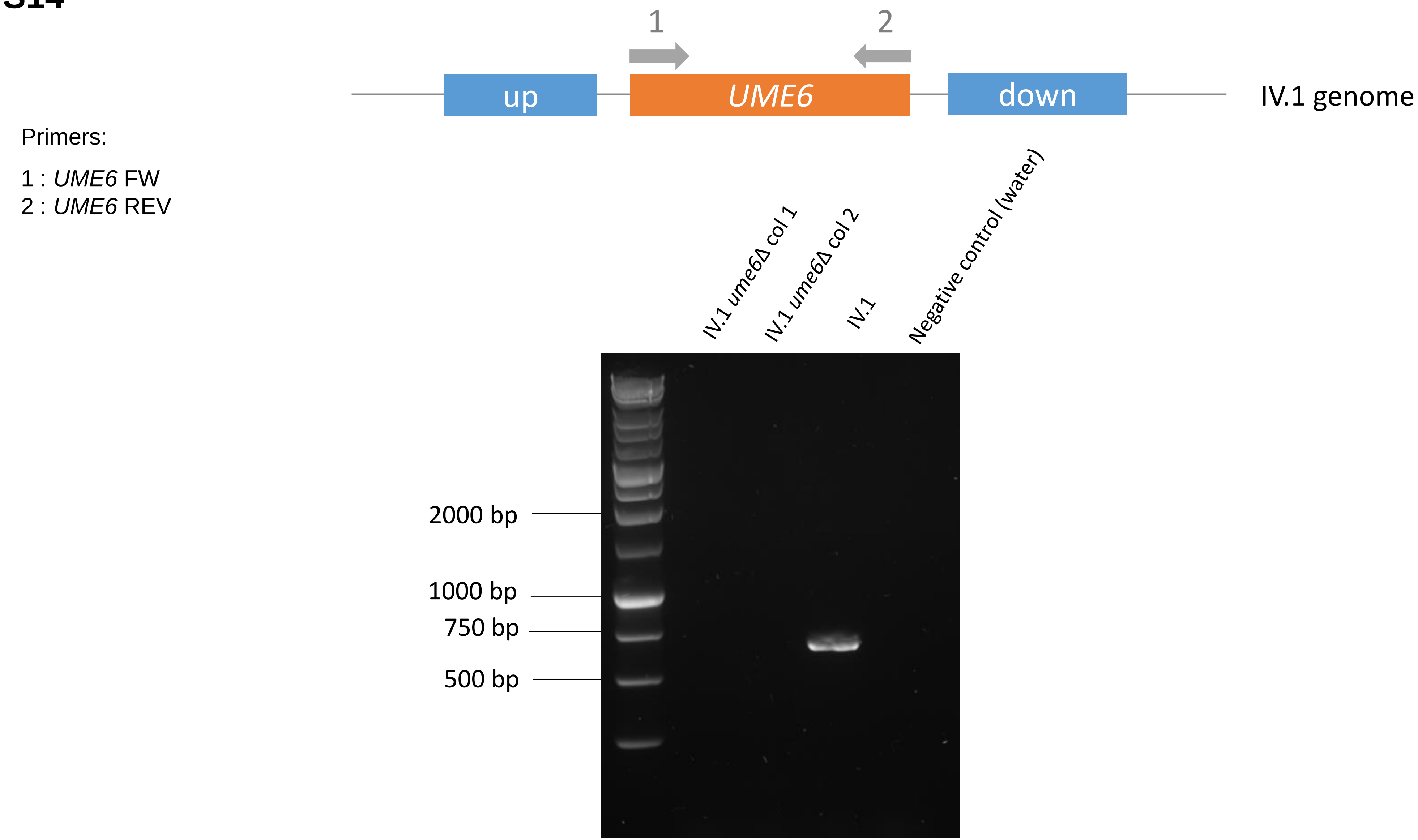

Verification of the deletion of *UME6* in the *ume6*Δ strain. An approximately 700 bp fragment located within the *UME6* gene was amplified. As shown on the electrophoresis gel, the PCR product was present in the IV.1 strain and absent in the mutant strain.

Figure S15

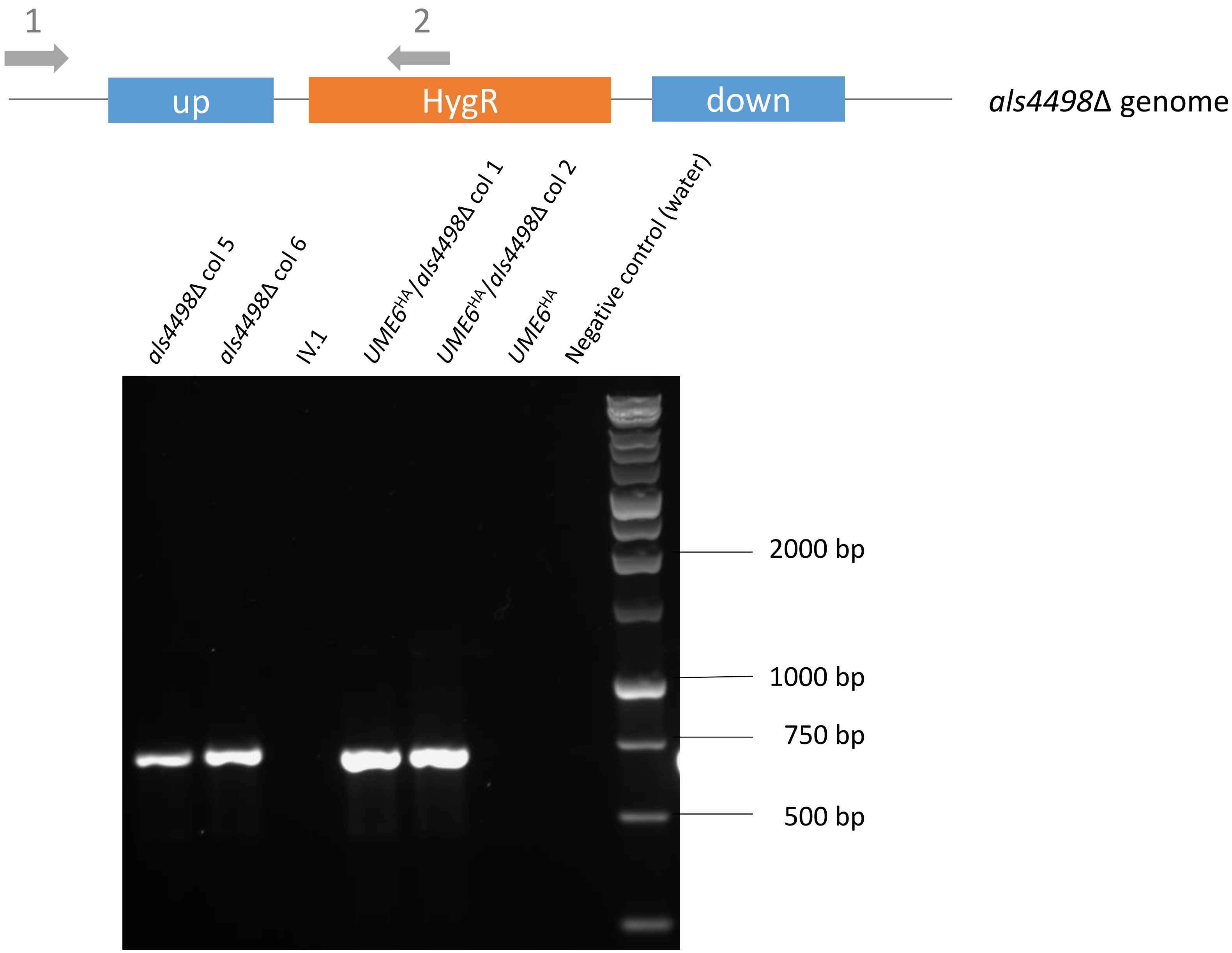

Primers:

1 : DEL *als4498* 5’ FW
2 : DEL *als4498* 5’ REV

Verification of the deletion of *ALS4498* in the *als4498*Δ and *UME6*^{HA}*/als4498*Δ strains. An approximately 700 bp fragment was amplified with a forward primer located upstream of the “up” region and a reverse primer in *HygR*. As shown on the electrophoresis gel, the PCR product was present in the mutant strain and absent in the IV.1 strain.

Figure S16

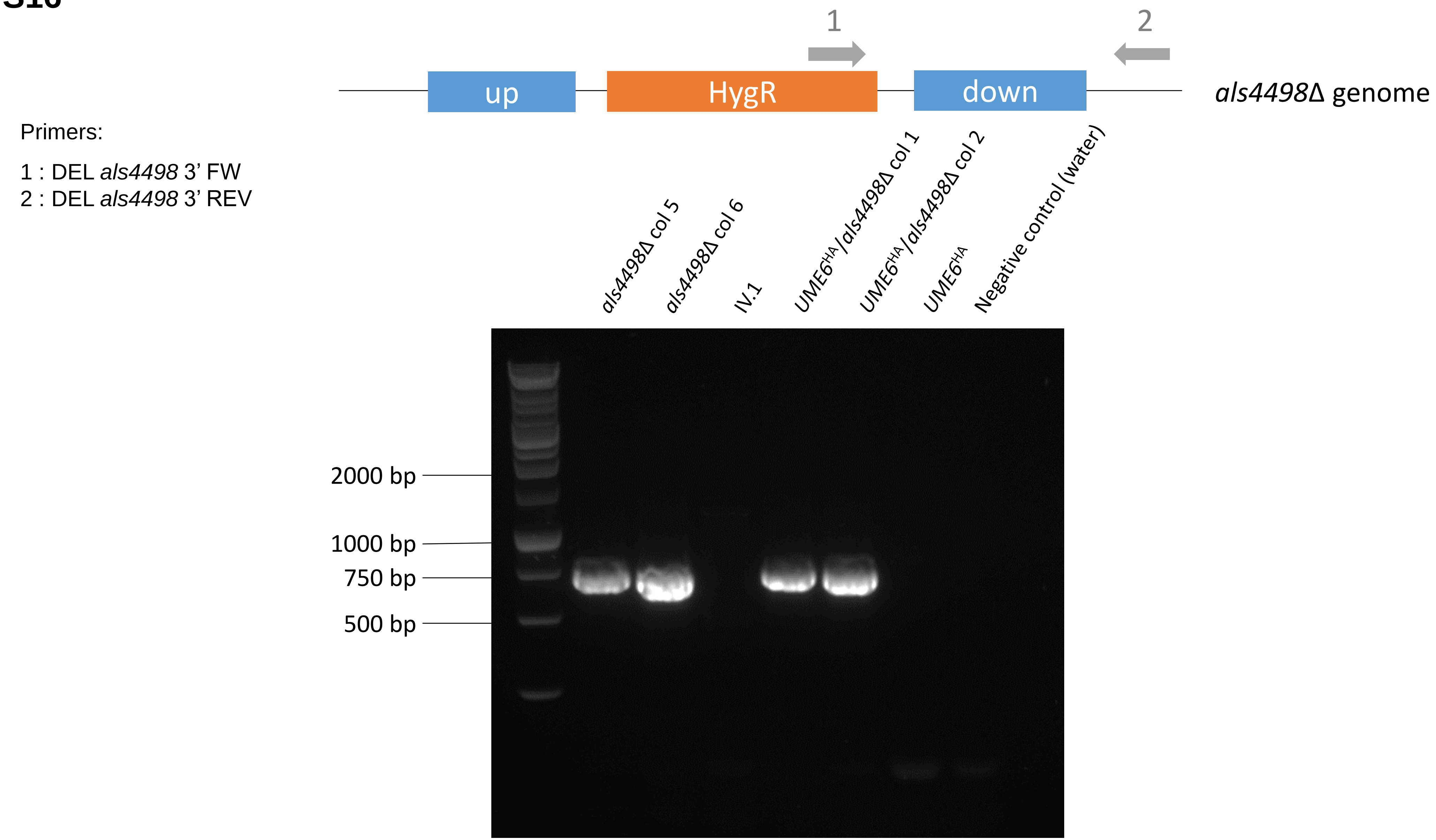

Verification of the deletion of *ALS4498* in the *als4498Δ* and *UME6^{HA}/als4498Δ* strains. An approximately 700 bp fragment was amplified with a forward primer in *HygR* and a reverse primer located downstream of the "down" region. As shown on the electrophoresis gel, the PCR product was present in the mutant strain and absent in the IV.1 strain.

Figure S17

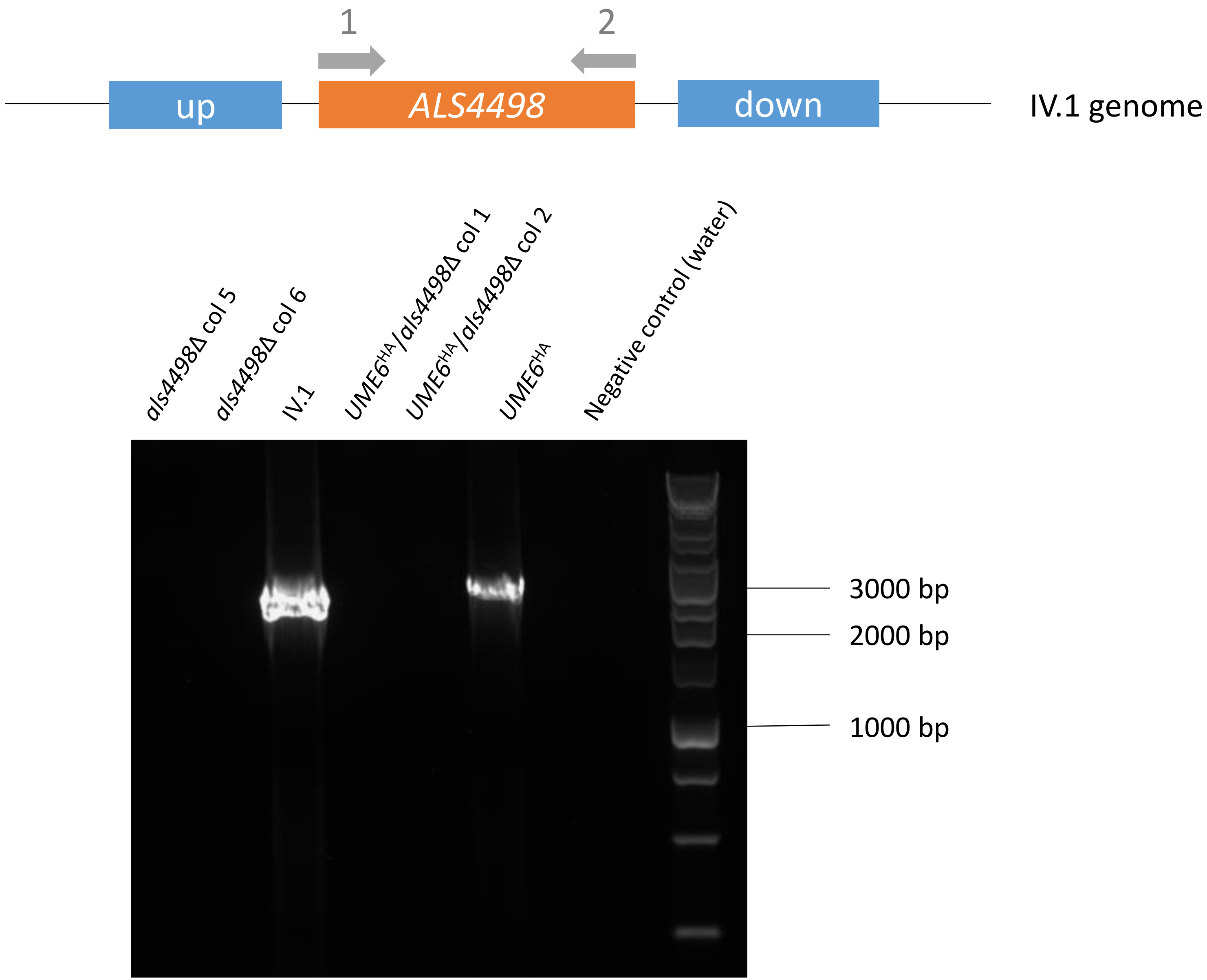

Primers:

1 : als4498 FW
2 : *als4498* REV

Verification of the deletion of *ALS4498* in the *als4498*Δ and *UME6^{HA}/als4498*Δ strains. An approximately 2500 bp fragment located within the *ALS4498* gene was amplified. As shown on the electrophoresis gel, the PCR product was present in the IV.1 strain and absent in the mutant strain.

Figure S18

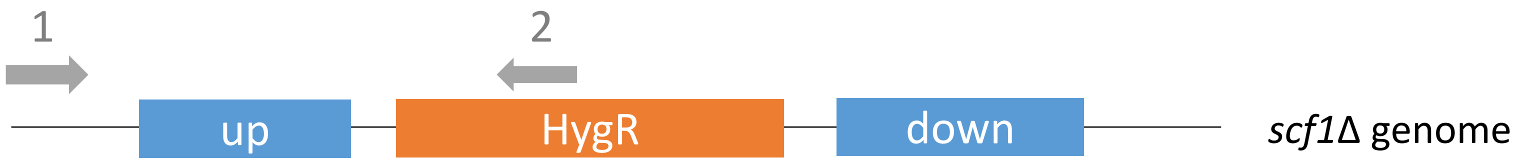

Primers:

1 : DEL *SCF1* 5’ FW
2 : DEL HygR 5’ REV

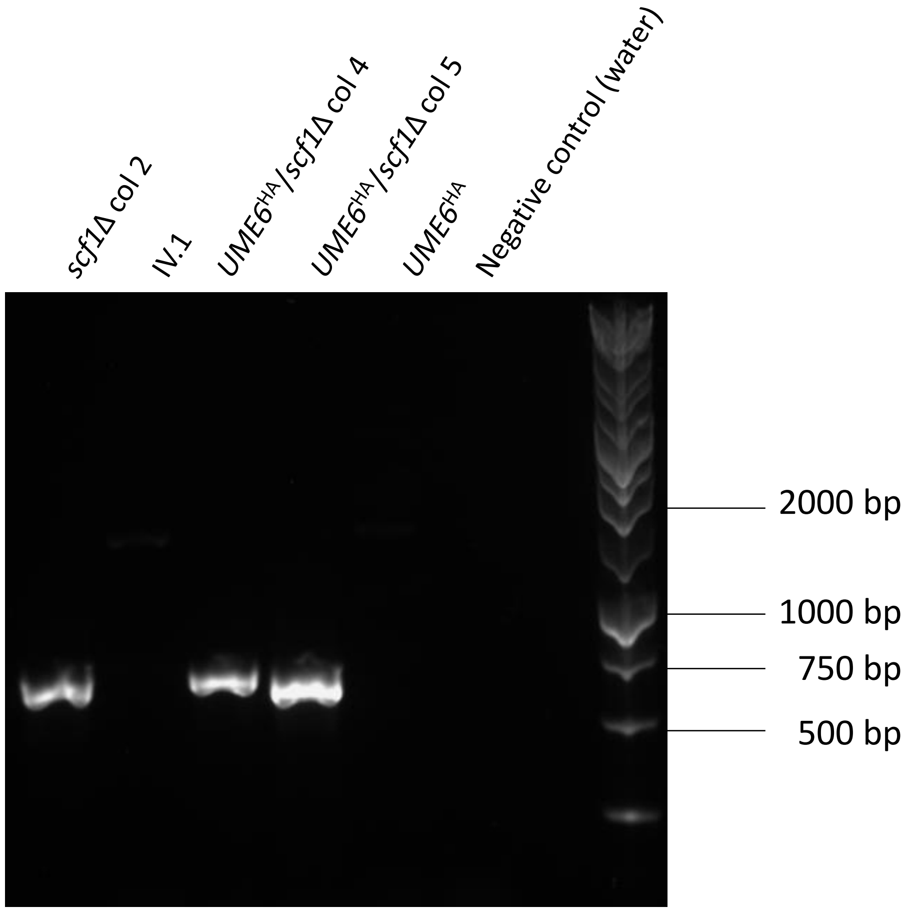

Verification of the deletion of *SCF1* in the *scf1*Δ and *UME6*^{HA}/*scf1*Δ strains. An approximately 700 bp fragment was amplified with a forward primer located upstream of the “up” region and a reverse primer in *HygR*. As shown on the electrophoresis gel, the PCR product was present in the mutant strain and absent in the IV.1 strain.

Figure S19

Primers:

1 : DEL HygR 3’ FW
2 : DEL *SCF1* 3’ REV

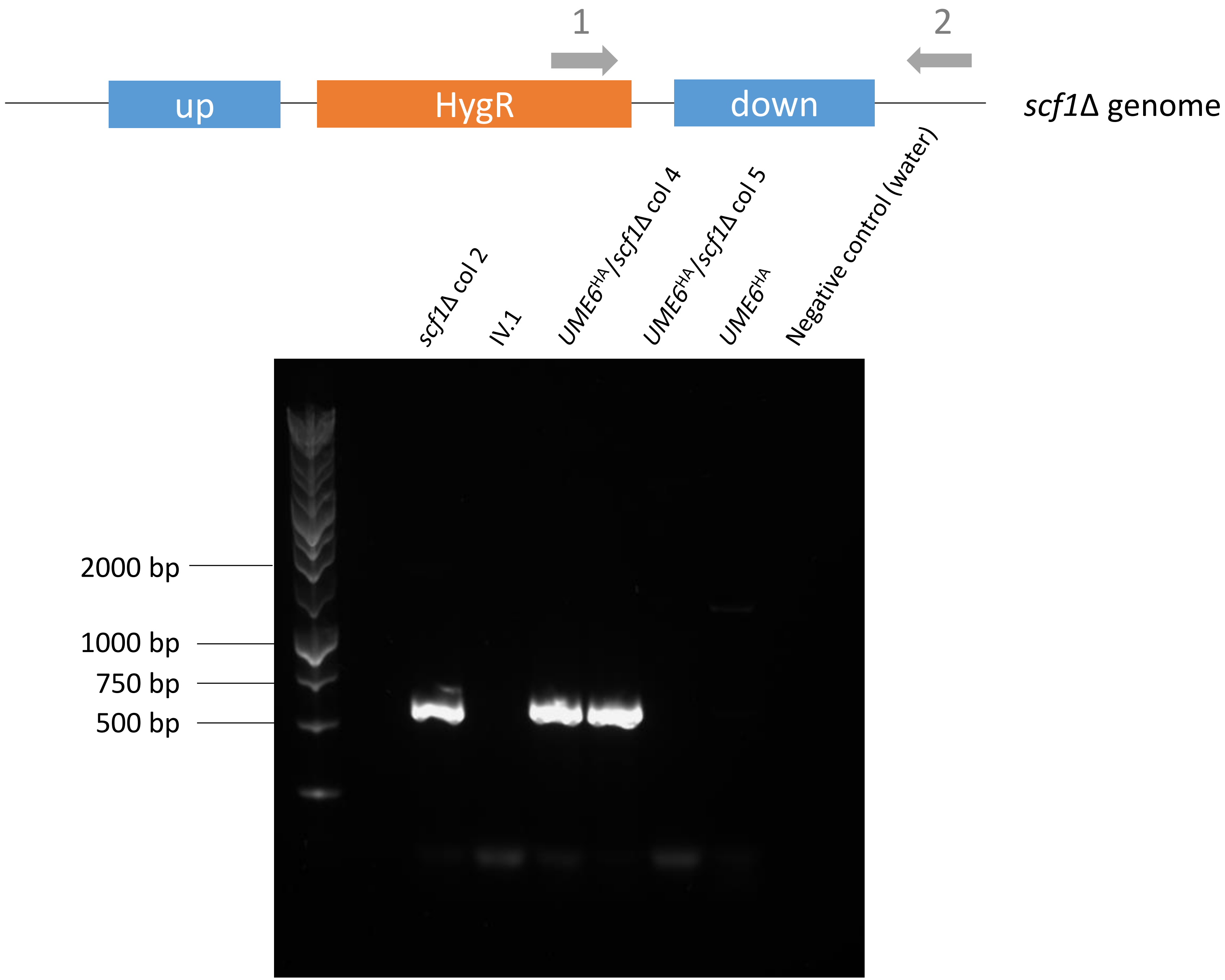

Verification of the deletion of *SCF1* in the *scf1*Δ and *UME6*^{HA}*/scf1*Δ strains. An approximately 700 bp fragment was amplified with a forward primer in *HygR* and a reverse primer located downstream of the “down” region. As shown on the electrophoresis gel, the PCR product was present in the mutant strain and absent in the IV.1 strain.

Figure S20

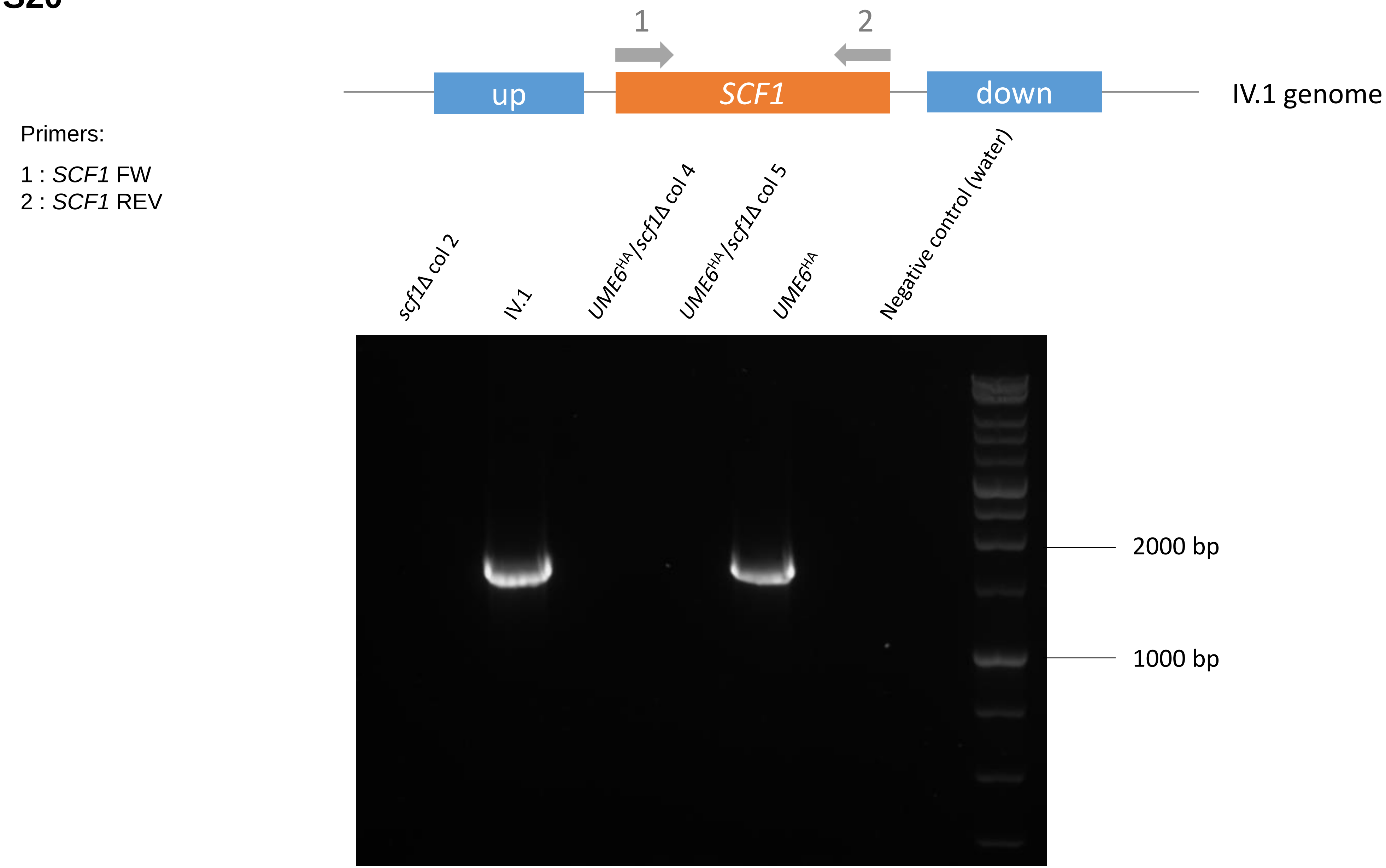

Verification of the deletion of *SCF1* in the *scf1*Δ and *UME6*^{HA}*/scf1*Δ strains. An approximately 1500 bp fragment located within the *SCF1* gene was amplified. As shown on the electrophoresis gel, the PCR product was present in the IV.1 strain and absent in the mutant strain.

Figure S21

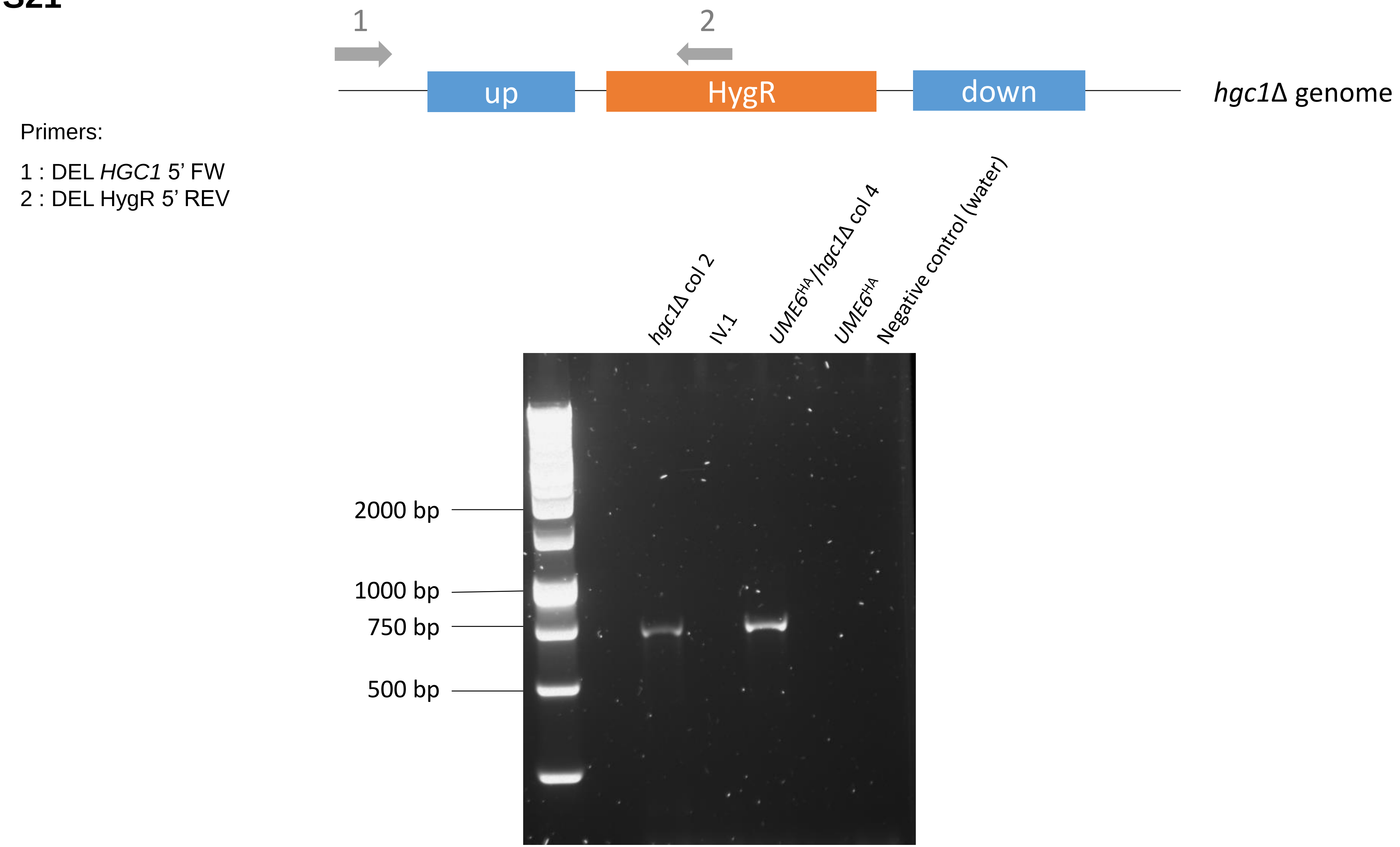

Verification of the deletion of *HGC1* in the *hgc1*Δ and *UME6*^{HA}*/hgc1*Δ strains. An approximately 700 bp fragment was amplified with a forward primer located upstream of the "up" region and a reverse primer in *HygR*. As shown on the electrophoresis gel, the PCR product was present in the mutant strain and absent in the IV.1 strain.

Figure S22

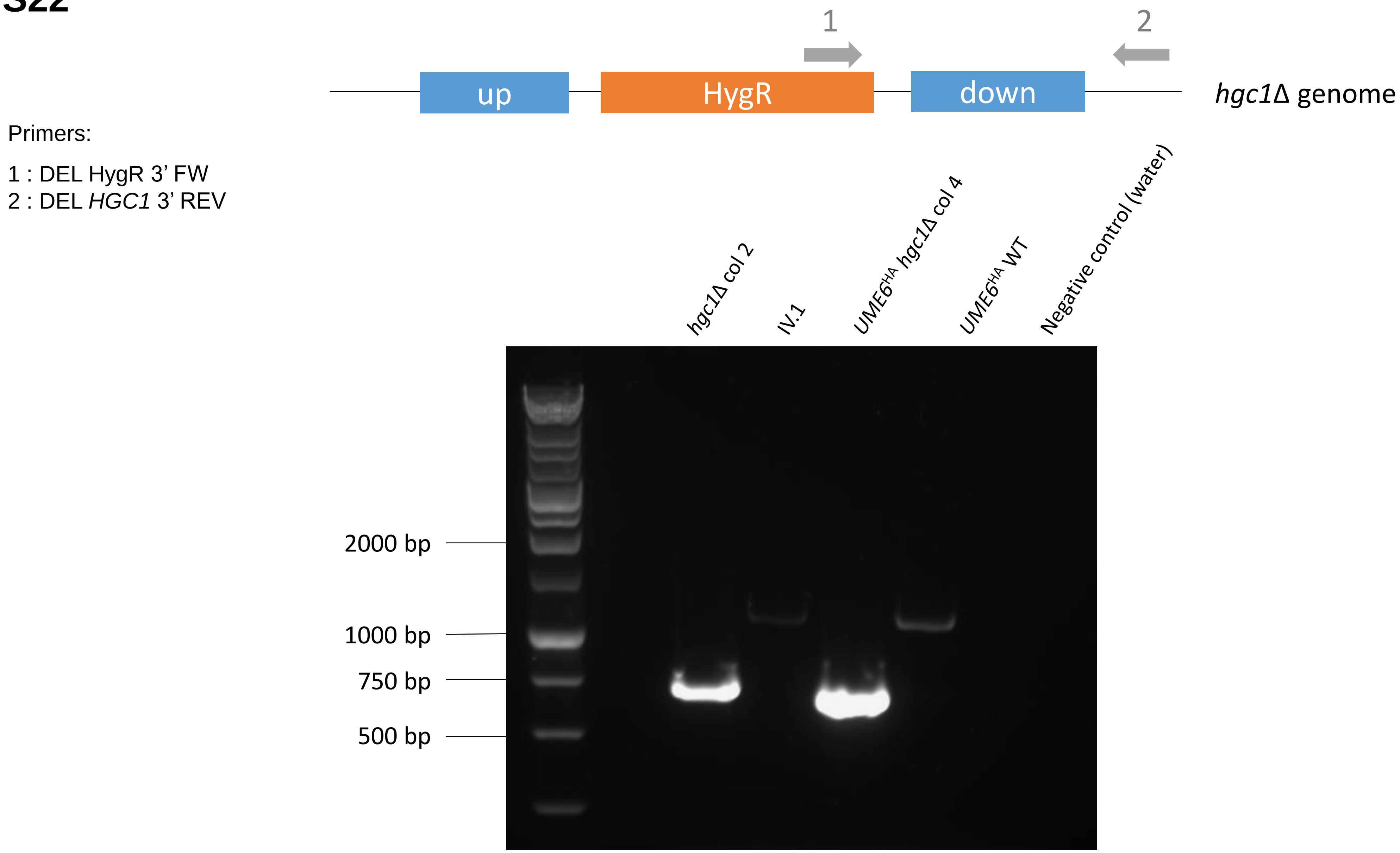

Verification of the deletion of *HGC1* in the *hgc1*Δ and *UME6*^{HA}*/hgc1*Δ strains. An approximately 700 bp fragment was amplified with a forward primer in *HygR* and a reverse primer located downstream of the "down" region. As shown on the electrophoresis gel, the PCR product was present in the mutant strain and absent in the IV.1 strain.

Figure S23

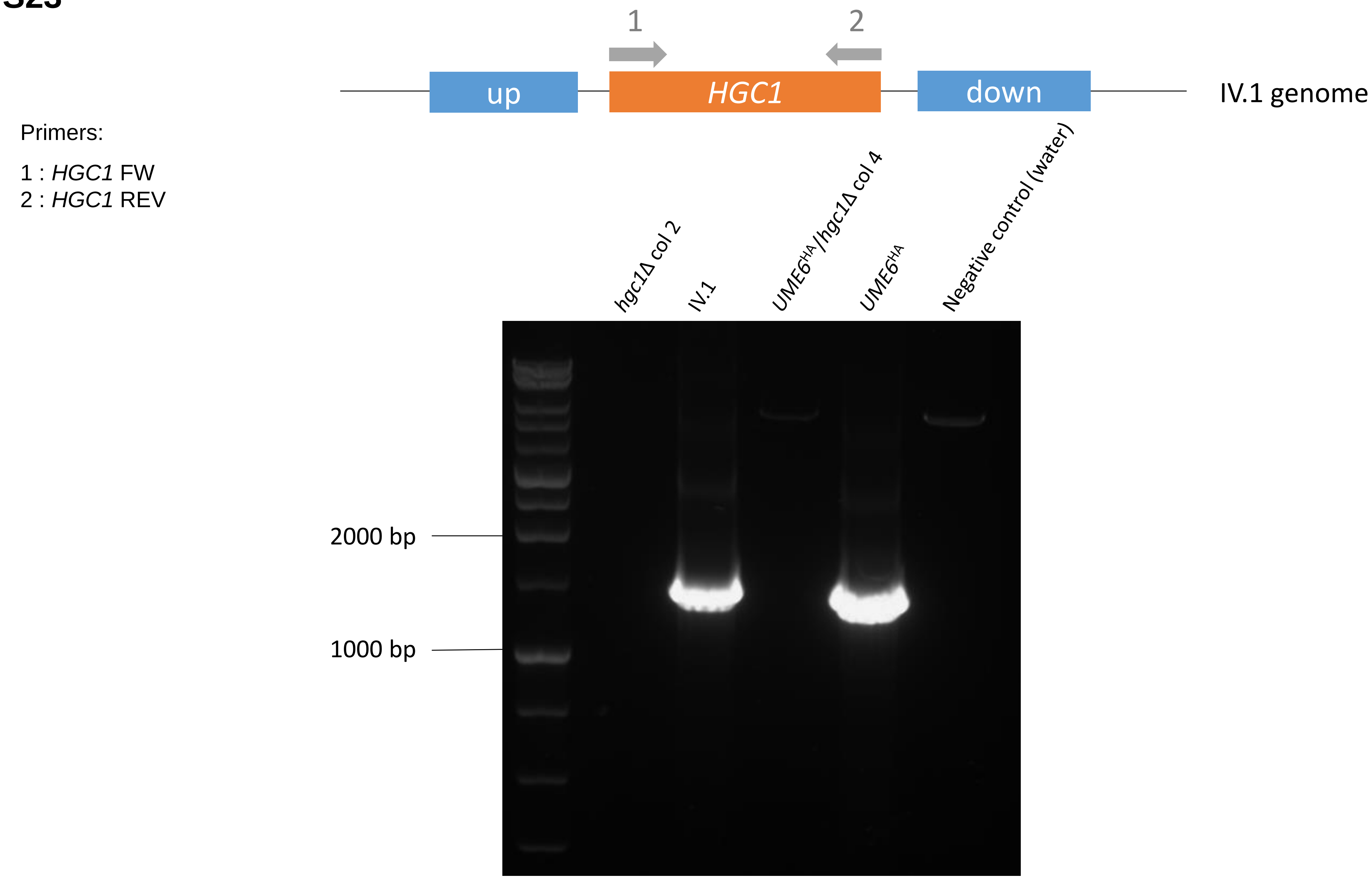

Verification of the deletion of *HGC1* in the *hgc1Δ* and *UME6^HA^/hgc1Δ* strains. An approximately 1500 bp fragment located within the *HGC1* gene was amplified. As shown on the electrophoresis gel, the PCR product was present in the IV.1 strain and absent in the mutant strain.

Figure S24

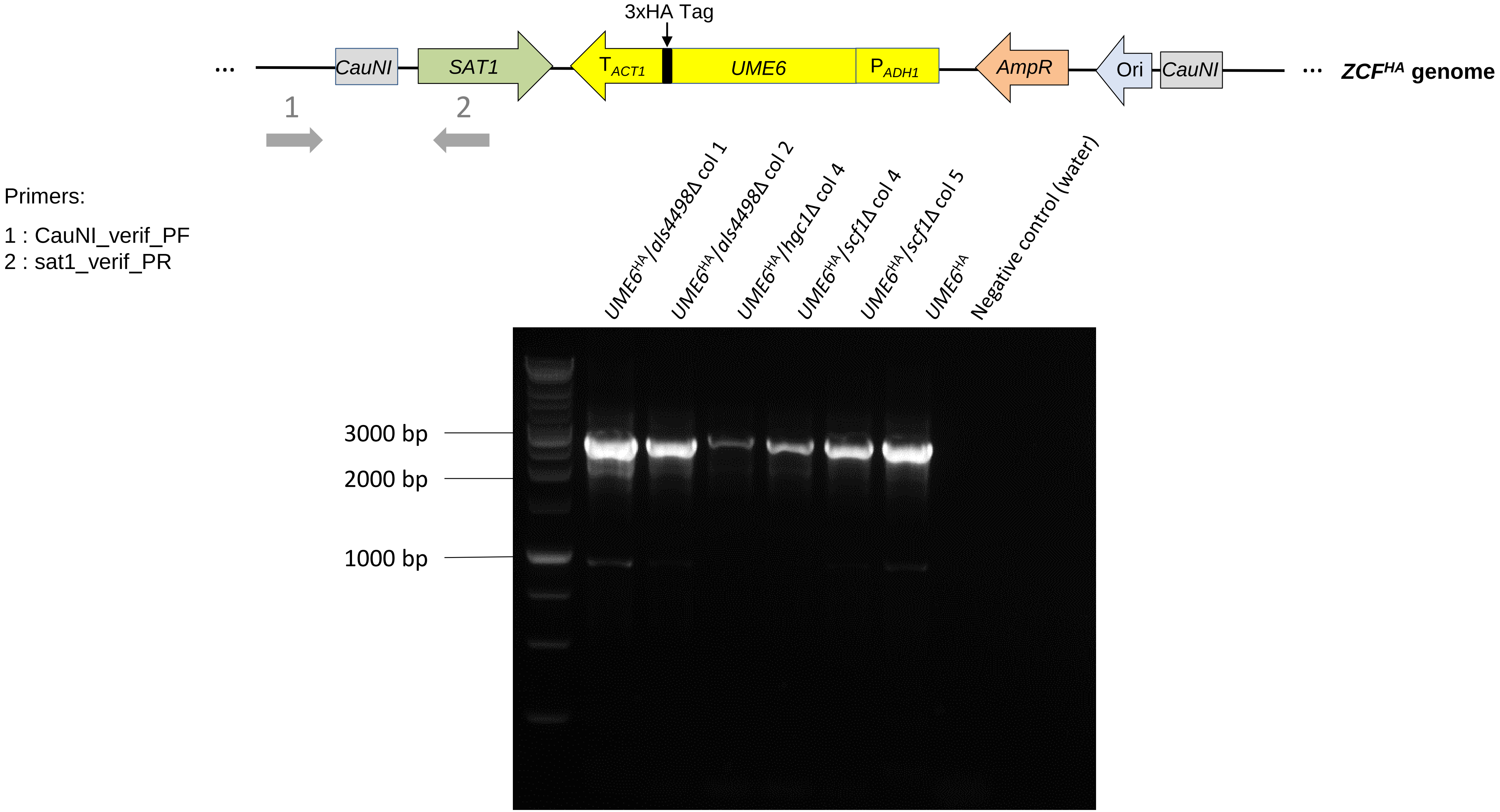

Verification of the presence of the transformation cassette in all the mutant strains constructed in the *UME6*^{HA} background. An approximately 2.5 kb fragment was amplified with a forward primer in the upstream region of the *C. auris* neutral site *CauNI* and a reverse primer in *SAT1.* As shown on the electrophoresis gel, the PCR product was present in the mutant strains and absent in the IV.1 strain.

Figure S25

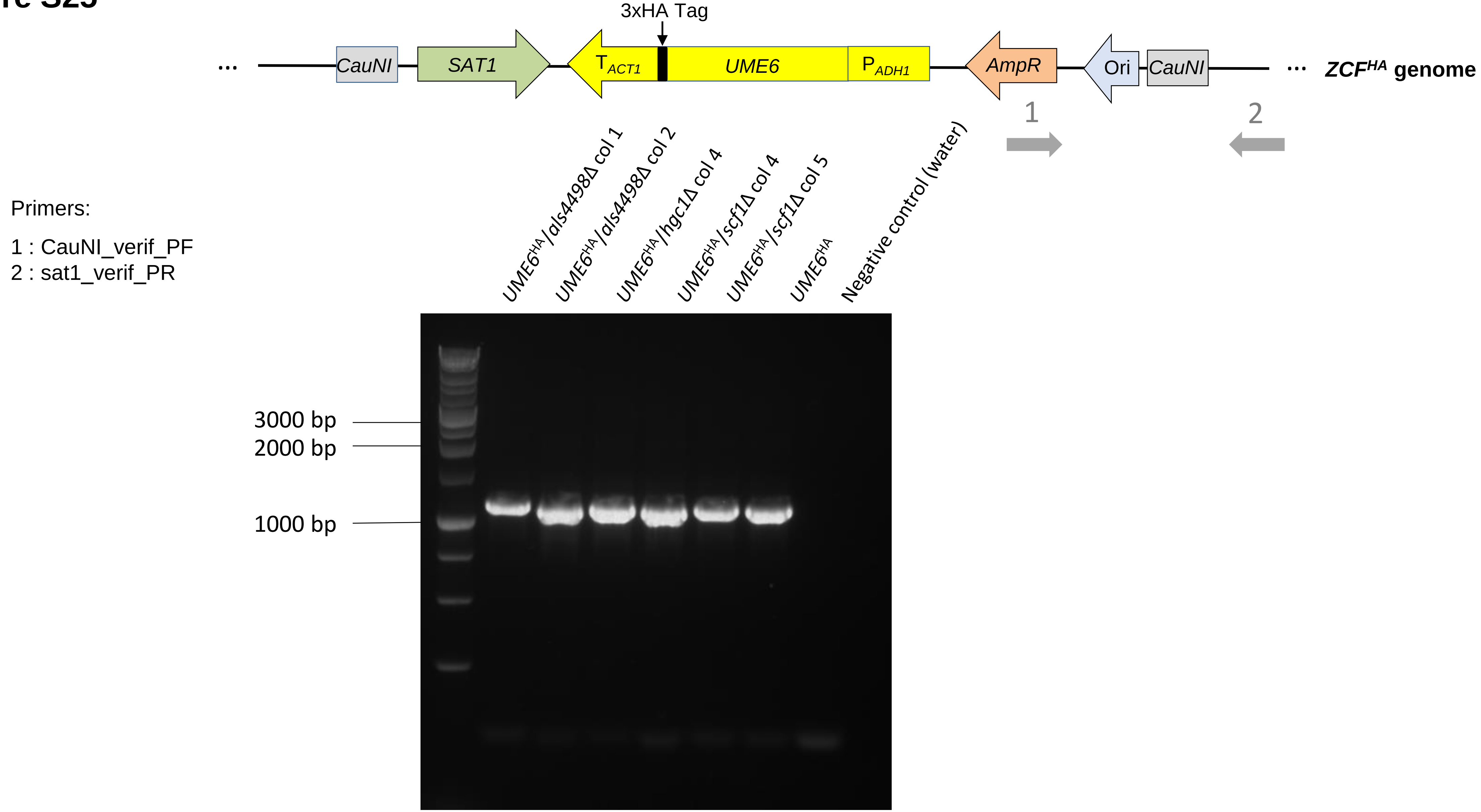

Verification of the presence of the transformation cassette in all the mutant strains constructed in the *UME6*^{HA} background. An approximately 1 kb fragment was amplified with a forward primer in the upstream region of the *C. auris* neutral site *CauNI* and a reverse primer in *SAT1.* As shown on the electrophoresis gel, the PCR product was present in the mutant strains and absent in the IV.1 strain.

Figure S26

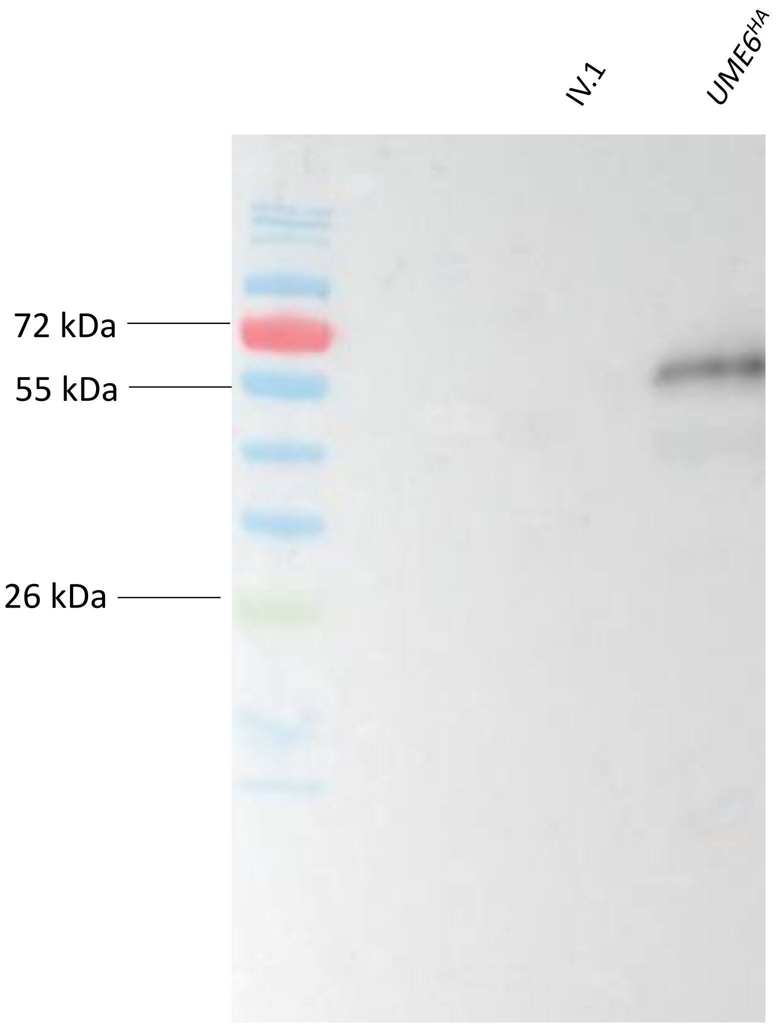

Verification of HA tagging of UME6p in the *UME6*^{HA} strain. Western blot analysis was performed using a HA tag monoclonal antibody. A band was detected at the expected size (approximately 50 kDa) in the *UME6*^{HA} strain and not in the IV.1 strain.

Figure S27

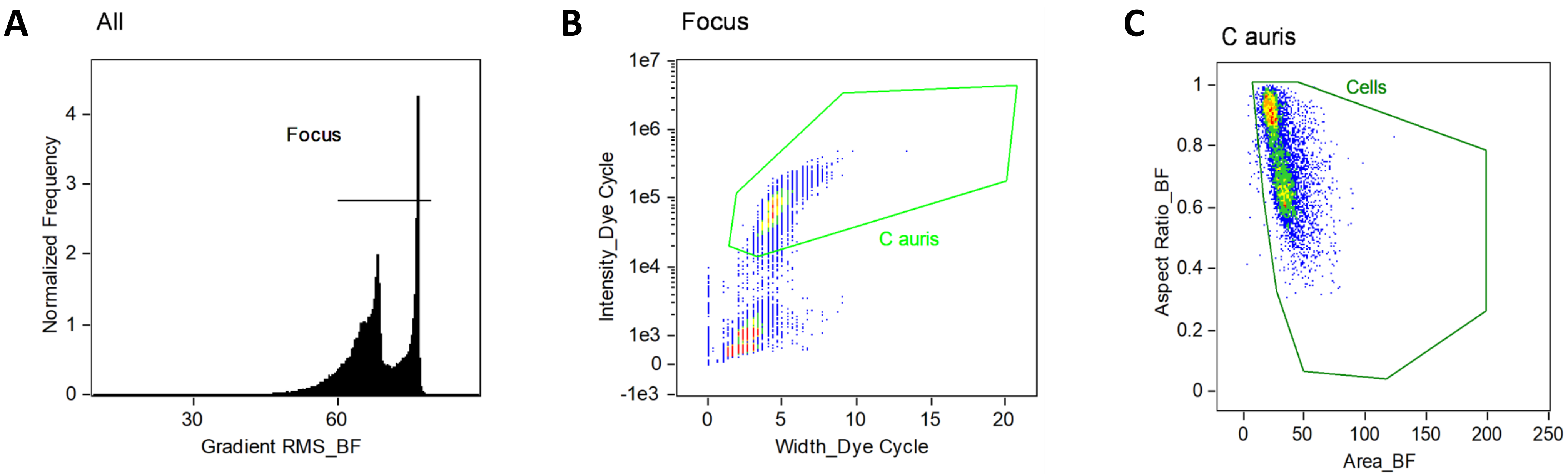

Gating strategy used for Image Flow Cytometry.

A. Cells were gated for the gradient root mean square (RMS) of the bright-field channel to exclude the out of focus events.

B. Candida auris cells were then identified by plotting DyeCycle™ Green width (measured in microns) versus DyeCycle™ Green fluorescence intensity.

C. After gating on the DyeCycle™ Green positive population, the area of the bright-field image (expressed in µm²) versus the aspect ratio (minor axis/major axis) were used to exclude cell debris and clumps.
