# Supplementary material for: Ume6-dependent pathways of morphogenesis and biofilm formation in Candida auris
Source: Microbiol Spectr. 2024 Sep 19;12(11):e01531-24. doi: 10.1128/spectrum.01531-24 (PMC11537075; doi:10.1128/spectrum.01531-24)
Supplement: Supplemental tables — Tables S1 to S3. [file spectrum.01531-24-s0005.docx]

**Table S1. Description of the *Candida auris* strains of this study.**

| **Name** | **Genotype** | **Parent** | **Reference** |
| --- | --- | --- | --- |
| IV.1 | Clinical isolate | - | [1] |
| *ume6*Δ | *ume6*Δ::*HygR* | IV.1 | This study |
| *als4498*Δ | *als4498*Δ::*HygR* | IV.1 | This study |
| *scf1*Δ | *scf1*Δ::*HygR* | IV.1 | This study |
| *hgc1*Δ | *hgc1*Δ::*HygR* | IV.1 | This study |
| *UME6^HA^* | P*_ADH1_*-*UME6*-3XHA Tag-T*_ACT1_*::*SAT1* | IV.1 | This study |
| *UME6^HA^ / als4498*Δ | P*_ADH1_*-*UME6*-3XHA Tag-T*_ACT1_*::*SAT1 als4498*Δ::*HygR* | *UME6^HA^* | This study |
| *UME6^HA^ / scf1*Δ | P*_ADH1_*-*UME6*-3XHA Tag-T*_ACT1_*::*SAT1 scf1*Δ::*HygR* | *UME6^HA^* | This study |
| *UME6^HA^ / hgc1*Δ | P*_ADH1_*-*UME6*-3XHA Tag-T*_ACT1_*::*SAT1 hgc1*Δ::*HygR* | *UME6^HA^* | This study |

**Table S2: List of the primers & RNA guides used for this study**

| **Name** | **Primer / RNA guide**  **& description** | **Sequence (5’ – 3’)** |
| --- | --- | --- |
| *UME6* up FW | Primer, construction of *UME6* deletion cassette | TAGCCTTTTCGCCTCTTCTCG |
| *UME6* up REV | Primer, construction of *UME6* deletion cassette | GTATTCTGGGCCTCCATGTCCCAGGAGTGGTAACGGTGTAA |
| NatR FW | Primer, construction of *UME6* deletion cassette | TTACACCGTTACCACTCCTGGGACATGGAGGCCCAGAATAC |
| NatR REV | Primer, construction of *UME6* deletion cassette | TCTGAGAGATGTAGTTACATTGGACAGTATAGCGACCAGCATTCAC |
| *UME6* down FW | Primer, construction of *UME6* deletion cassette | GTGAATGCTGGTCGCTATACTGTCCAATGTAACTACATCTCTCAGA |
| *UME6* down REV | Primer, construction of *UME6* deletion cassette | CCGTATTCTAGGGATGTCTCAC |
| Fusion PCR finale del *UME6* FW | Primer, construction of *UME6* deletion cassette | TCACCGACTTTTTCAAGGCAACC |
| Fusion PCR finale del *UME6* REV | Primer, construction of *UME6* deletion cassette | TTTACGTCATTCGGCTGTGGAG |
| *UME6* 5’ FW | Primer, *UME6* deletion verification | CAACACCTTTCCTTGTTTTCACG |
| *UME6* 5’ REV | Primer, *UME6* deletion verification | TGTCAAGGAGGGTATTCTGGG |
| *UME6* 3’ FW | Primer, *UME6* deletion verification | ATGCGTCAATCGTATGTGAATGCT |
| *UME6* 3’ REV | Primer, *UME6* deletion verification | CTCATCGAGCACTTGGCAAACA |
| *UME6* FW | Primer, *UME6* deletion verification | AGTTTTGCAAGGCAGGATATGTTTG |
| *UME6* REV | Primer, *UME6* deletion verification | TTGGCCAGATCTGGCCTTAAG |
| *ALS33* up FW | Primer, construction of *ALS33* deletion cassette | TTTAGTCTACCGGCTCAACGC |
| *ALS33* up REV | Primer, construction of *ALS33* deletion cassette | AATATCGAACAGCAAGCACTATTGTGGGTACCAGTATCTTGGG |
| HygR del *ALS33* FW | Primer, construction of *ALS33* deletion cassette | CCCAAGATACTGGTACCCACAATAGTGCTTGCTGTTCGATATT |
| HygR del *ALS33* REV | Primer, construction of *ALS33* deletion cassette | GGCGAGTCCAAAGAAAGTAAAACAATTTTATGATGGAATGAATGGGATG |
| *ALS33* down FW | Primer, construction of *ALS33* deletion cassette | CATCCCATTCATTCCATCATAAAATTGTTTTACTTTCTTTGGACTCGCC |
| *ALS33* down REV | Primer, construction of *ALS33* deletion cassette | GCCATTGAAGGATCTGAATTGGC |
| Fusion PCR finale del *ALS33* FW | Primer, construction of *ALS33* deletion cassette | GACGAAAACATACCTCGTCAATCAA |
| Fusion PCR finale del *ALS33* REV | Primer, construction of *ALS33* deletion cassette | AGAGCCGTGTGTATGTTGTCTAA |
| DEL *ALS33* 5’ FW | Primer, *ALS33* deletion verification | GAATCTCGTAGAATCTAAGCCTAGA |
| DEL *ALS33* 5’ REV | Primer, *ALS33* deletion verification | TTCCATTTTAGGCGTGAGGTAATC |
| DEL *ALS33* 3’ FW | Primer, *ALS33* deletion verification | ATAAAATAGCCACCCAAGGCATTTC |
| DEL *ALS33* 3’ REV | Primer, *ALS33* deletion verification | AGTTGGTCGACTGGTGTCCTA |
| *ALS33* FW | Primer, *ALS33* deletion verification | GTTCTTGCTTCGTTCTTGTTGGG |
| *ALS33* REV | Primer, *ALS33* deletion verification | CAGAAAAGCTTGCAGTGGAGC |
| *SCF1* up FW | Primer, construction of *SCF1* deletion cassette | CGGCTTACAATGCCCAGAATG |
| *SCF1* up REV | Primer, construction of *SCF1* deletion cassette | TATCGAACAGCAAGCACTATACGTGGAGGTGAAGTTTTAAGATAGAG |
| HygR del *SCF1* FW | Primer, construction of *SCF1* deletion cassette | CTCTATCTTAAAACTTCACCTCCACGTATAGTGCTTGCTGTTCGATA |
| HygR del *SCF1* REV | Primer, construction of *SCF1* deletion cassette | CACTAGGTCCACTTGGTCCTATTTTATGATGGAATGAATGGGATG |
| *SCF1* down FW | Primer, construction of *SCF1* deletion cassette | CATCCCATTCATTCCATCATAAAATAGGACCAAGTGGACCTAGTG |
| *SCF1* down REV | Primer, construction of *SCF1* deletion cassette | TGAGATACACTAGCAGAGGGTC |
| Fusion PCR finale del *SCF1* FW | Primer, construction of *SCF1* deletion cassette | CACAGGAAGAACCACTTTCACG |
| Fusion PCR finale del *SCF1* REV | Primer, construction of *SCF1* deletion cassette | AACTTGGGAGGCAGAAGGAG |
| DEL *SCF1* 5’ FW | Primer, *SCF1* deletion verification | AGGTCTCTCACTGGGAACTTG |
| DEL HygR 5’ REV | Primer, *SCF1* deletion verification | TTCCATTTTAGGCGTGAGGTAATC |
| DEL HygR 3’ FW | Primer, *SCF1* deletion verification | ATAAAATAGCCACCCAAGGCATTTC |
| DEL *SCF1* 3’ REV | Primer, *SCF1* deletion verification | AACAAGGCAGCAAGTAAAGCGC |
| *SCF1* FW | Primer, *SCF1* deletion verification | TCTTGCAGGTGTAGTGGCTG |
| *SCF1* REV | Primer, *SCF1* deletion verification | GCTGTACTAGTTGAACCGGTAATT |
| *HGC1* up FW | Primer, construction of *HGC1* deletion cassette | TCTCTGCCTCTCTGCCTCTTTT |
| *HGC1* up REV | Primer, construction of *HGC1* deletion cassette | TATCGAACAGCAAGCACTATACGGTGGGGGTGGTCCCTT |
| HygR del *HGC1* FW | Primer, construction of *HGC1* deletion cassette | AAGGGACCACCCCCACCGTATAGTGCTTGCTGTTCGATA |
| HygR del *HGC1* REV | Primer, construction of *HGC1* deletion cassette | GGGCATGTGGCGTTAATAAATTTCATTTTATGATGGAATGAATGGGATG |
| *HGC1* down FW | Primer, construction of *HGC1* deletion cassette | CATCCCATTCATTCCATCATAAAATGAAATTTATTAACGCCACATGCCC |
| *HGC1* down REV | Primer, construction of *HGC1* deletion cassette | ATGTCGCTTCTGGCAGCTTCAT |
| Fusion PCR finale del *HGC1* FW | Primer, construction of *HGC1* deletion cassette | CCTCGTTTCGCGCACAATCT |
| Fusion PCR finale del *HGC1* REV | Primer, construction of *HGC1* deletion cassette | GGCATTAATCTCGTCTAACTTCAGA |
| DEL *HGC1* 5’ FW | Primer, *HGC1* deletion verification | CAAATCTGGCTTACCGCTTGCT |
| DEL *HGC1* 3’ REV | Primer, *HGC1* deletion verification | CAGTGAACCCCTGTTATTTTCACC |
| *HGC1* FW | Primer, *HGC1* deletion verification | ATGTCCATCCATGGCACCTTTTC |
| *HGC1* REV | Primer, *HGC1* deletion verification | CGACTTGTCGTCCTCTATGCT |
| RNAg *UME6* 5’ | RNA guide, *UME6* deletion | ACGGGGTCAGCAAAGATGTC |
| RNAg *UME6* 3’ | RNA guide, *UME6* deletion | TCGATGAGCTCTCTACTAAG |
| RNAg *ALS33* 5’ | RNA guide, *ALS33* deletion | GTTCTCGGGTGATAACGTGG |
| RNAg *ALS33* 3’ | RNA guide, *ALS33* deletion | GGACCAATGAAGAAGCAGAG |
| RNAg *SCF1* 5’ | RNA guide, *SCF1* deletion | GTCGTCGTCAGCTGGCAAGG |
| RNAg *SCF1* 3’ | RNA guide, *SCF1* deletion | CCACAACCAAGTGGGCCTAG |
| RNAg *HGC1* 5’ | RNA guide, *HGC1* deletion | ACCAGTACAAGTACCCCCAT |
| RNAg *HGC1* 3’ | RNA guide, *HGC1* deletion | GTCGTCCTCTATGCTGATTG |

**Table S3. Adhesins of *Candida auris***

**Hyr / Iff family**

| **B9J08 locus** | **SBP28 locus** | **Reference** |
| --- | --- | --- |
| B9J08_001531 (*IFF4*) | SBP28_001903 | [2, 3] |
| B9J08_004098 | SBP28_000003 | [2, 3] |
| B9J08_004109 | SBP28_005093 | [2, 3] |
| B9J08_004892 | SBP28_004302 | [2, 3] |
| B9J08_004100 (*HYR3*) | SBP28_000001 | [2, 3] |
| B9J08_004110 (*IFF9*) | SBP28_005092 | [2, 3] |
| B9J08_001155 | SBP28_003313 | [2, 3] |
| B9J08_004451 | SBP28_004759 | [2, 3] |

**Als family**

| **B9J08 locus** | **SBP28 locus** | **Reference** |
| --- | --- | --- |
| B9J08_002582 (*ALS7*) | SBP28_005429 | [2] |
| B9J08_004498 (*ALS1*) | SBP28_004635 | [2] |
| B9J08_004112 (*ALS4*) | SBP28_005090 | [2] |

**Scf family**

| **B9J08 locus** | **SBP28 locus** | **Reference** |
| --- | --- | --- |
| B9J08_001458 (SCF1) | SBP28_003606 | [4] |

**References**

1. Theill L, Dudiuk C, Morales-Lopez S, Berrio I, Rodriguez JY, Marin A, Gamarra S, Garcia-Effron G. 2018. Single-tube classical PCR for Candida auris and Candida haemulonii identification. Rev Iberoam Micol 35:110-2. <https://doi.org/10.1016/j.riam.2018.01.003>

2. Munoz JF, Welsh RM, Shea T, Batra D, Gade L, Howard D, Rowe LA, Meis JF, Litvintseva AP, Cuomo CA. 2021. Clade-specific chromosomal rearrangements and loss of subtelomeric adhesins in Candida auris. Genetics 218. <https://doi.org/10.1093/genetics/iyab029>

3. Smoak RA, Snyder LF, Fassler JS, He BZ. 2023. Parallel expansion and divergence of an adhesin family in pathogenic yeasts. Genetics 223. <https://doi.org/10.1093/genetics/iyad024>

4. Santana DJ, Anku JAE, Zhao G, Zarnowski R, Johnson CJ, Hautau H, Visser ND, Ibrahim AS, Andes D, Nett JE, Singh S, O'Meara TR. 2023. A Candida auris-specific adhesin, Scf1, governs surface association, colonization, and virulence. Science 381:1461-7. <https://doi.org/10.1126/science.adf8972>
